# Efficient organized colorectal cancer screening in Shenzhen: a microsimulation modelling study

**DOI:** 10.1186/s12889-024-18201-w

**Published:** 2024-03-01

**Authors:** Minmin Zhu, Xuan Zhong, Tong Liao, Xiaolin Peng, Lin Lei, Ji Peng, Yong Cao

**Affiliations:** 1 Shenzhen Nanshan Center for Chronic Disease Control, Shenzhen City, 518054 Guangdong China; 2 Harbin Institute of Technology Shenzhen, Shenzhen City, Guangdong China; 3 Shenzhen Center for Chronic Disease Control, Shenzhen City, Guangdong China

**Keywords:** Microsimulation model, Colorectal cancer, Screening, Fecal immunochemical test, Risk assessment tool, Shenzhen

## Abstract

**Background:**

Colorectal cancer (CRC) is a global health issue with noticeably high incidence and mortality. Microsimulation models offer a time-efficient method to dynamically analyze multiple screening strategies. The study aimed to identify the efficient organized CRC screening strategies for Shenzhen City.

**Methods:**

A microsimulation model named CMOST was employed to simulate CRC screening among 1 million people without migration in Shenzhen, with two CRC developing pathways and real-world participation rates. Initial screening included the National Colorectal Polyp Care score (NCPCS), fecal immunochemical test (FIT), and risk-stratification model (RS model), followed by diagnostic colonoscopy for positive results. Several start-ages (40, 45, 50 years), stop-ages (70, 75, 80 years), and screening intervals (annual, biennial, triennial) were assessed for each strategy. The efficiency of CRC screening was assessed by number of colonoscopies versus life-years gained (LYG).

**Results:**

The screening strategies reduced CRC lifetime incidence by 14–27 cases (30.9–59.0%) and mortality by 7–12 deaths (41.5–71.3%), yielded 83–155 LYG, while requiring 920 to 5901 colonoscopies per 1000 individuals. Out of 81 screening, 23 strategies were estimated efficient. Most of the efficient screening strategies started at age 40 (17 out of 23 strategies) and stopped at age 70 (13 out of 23 strategies). Predominant screening intervals identified were annual for NCPCS, biennial for FIT, and triennial for RS models. The incremental colonoscopies to LYG ratios of efficient screening increased with shorter intervals within the same test category. Compared with no screening, when screening at the same start-to-stop age and interval, the additional colonoscopies per LYG increased progressively for FIT, NCPCS and RS model.

**Conclusion:**

This study identifies efficient CRC screening strategies for the average-risk population in Shenzhen. Most efficient screening strategies indeed start at age 40, but the optimal starting age depends on the chosen willingness-to-pay threshold. Within insufficient colonoscopy resources, efficient FIT and NCPCS screening strategies might be CRC initial screening strategies. We acknowledged the age-dependency bias of the results with NCPCS and RS.

**Supplementary Information:**

The online version contains supplementary material available at 10.1186/s12889-024-18201-w.

## Background

Colorectal cancer (CRC) is a global health issue, with noticeably high incidence and mortality [1]. In 2020, CRC was the second-highest newly diagnosed cancer (555 thousand) and the fifth-leading cause of cancer-related deaths in China (286 thousand) [2]. The incidence and mortality rate of CRC in China has been steadily increasing due to lifestyle changes and an ageing population [3]. Screening has been proven effective to reduce CRC incidence and mortality [4, 5], and was incorporated into public health services in some provinces of China, such as Cancer Screening Program in Urban Areas [6]. However, there are still barriers between CRC screening objectives and reality in China, such as large target population, restrained healthcare resources, and low participation rate of colonoscopy screening.

Shenzhen locates in the Guangdong Province of China and is one of the most developed and modern cities in China, with a population of approximately 17.7 million people in 2022 [7]. Despite its young population, Shenzhen is experiencing an increase in the incidence of CRC. From 2001 to 2018, the age-adjusted CRC incidence rate of local citizens increased from 22.87 to 29.99 per 100,000, even exceeding the national average in 2015 (28.63 vs 17.81 per 100,000) [8]. In response, a pilot CRC screening using a risk assessment questionnaire and fecal immunochemical test (FIT) was initiated in Shenzhen in 2020, but with limited coverage of only 50,000 individuals aged 45 to 74 years per year and without repeated screening. Therefore, studies on efficient CRC screening strategies are needed to reduce the disease burden.

Microsimulation models offer a time-efficient method to dynamically analyze combined intervention strategies, compared with large-scale randomized control trials or cohort studies that require more resources and longer durations. In some countries, microsimulation models have been adopted to evaluate the effect of CRC screening strategies [9–13]. Based on microsimulation models, several CRC screening guidelines have been suggested or updated [14, 15]. In Shanghai city of China, Cenin et al [16] and Wang et al [17] recommended using a validated FIT to substitute the current FIT screening, based on MISCAN-Colon modelling studies.

The objective of this study is to identify the efficient organized colorectal cancer screening strategies for Shenzhen City. Using the “Colon Modeling Open Simulation Tool” (CMOST) microsimulation model [18, 19], which contains two CRC developing pathways, we assessed the performance of diverse characteristics of screening strategies with real-world participation rates among average-risk individuals, including start-age, stop-age, and intervals of screening.

## Methods

We employed the CMOST model to simulate a birth cohort in Shenzhen, comparing various screening strategies with the no-screening scenario in this simulated population to identify efficient screening strategies. Aligning with current CRC screening practices in China, the two-stepped screening strategies in this paper involved a questionnaire-based risk assessment tool or FIT, or a combination of both, followed by a diagnostic colonoscopy for high-risk individuals identified during the initial step.

### CMOST

The CMOST is a well-documented and open-source microsimulation model used to evaluate the (cost-) effectiveness of CRC screening, in which the simulation results compared well with a large randomized controlled trial in reduction of CRC incidence and mortality, and agreed with two other intervention studies [18]. The model contains three main modules: the natural history of CRC, tools for CRC screening, and screening plans (start-age, stop-age, and interval). In CMOST, cancer manifests directly without adenomatous precursors in the Serrated-Cancer pathway, while it involves the sequential stages of early adenomas, advanced adenomas, preclinical cancer, and cancer in the Adenoma-Carcinoma pathway. There were three initial CMOST models with mean dwell times of 8 (CMOST8), 13 (CMOST13), and 19 years (CMOST19), respectively. And similar results can be achieved with models using different dwell times after calibration. The CMOST supports various screening interventions, such as colonoscopy, rectosigmoidoscopy, guaiac fecal occult blood test (gFOBT), FIT and other tests. The model also supports surveillance colonoscopy after the detection and removal of adenomas, with the implemented surveillance structure detailed in supplemental file 1. The source code of CMOST is available at https://github.com/poljan/CMOSTv2.

**Fig. 1:**
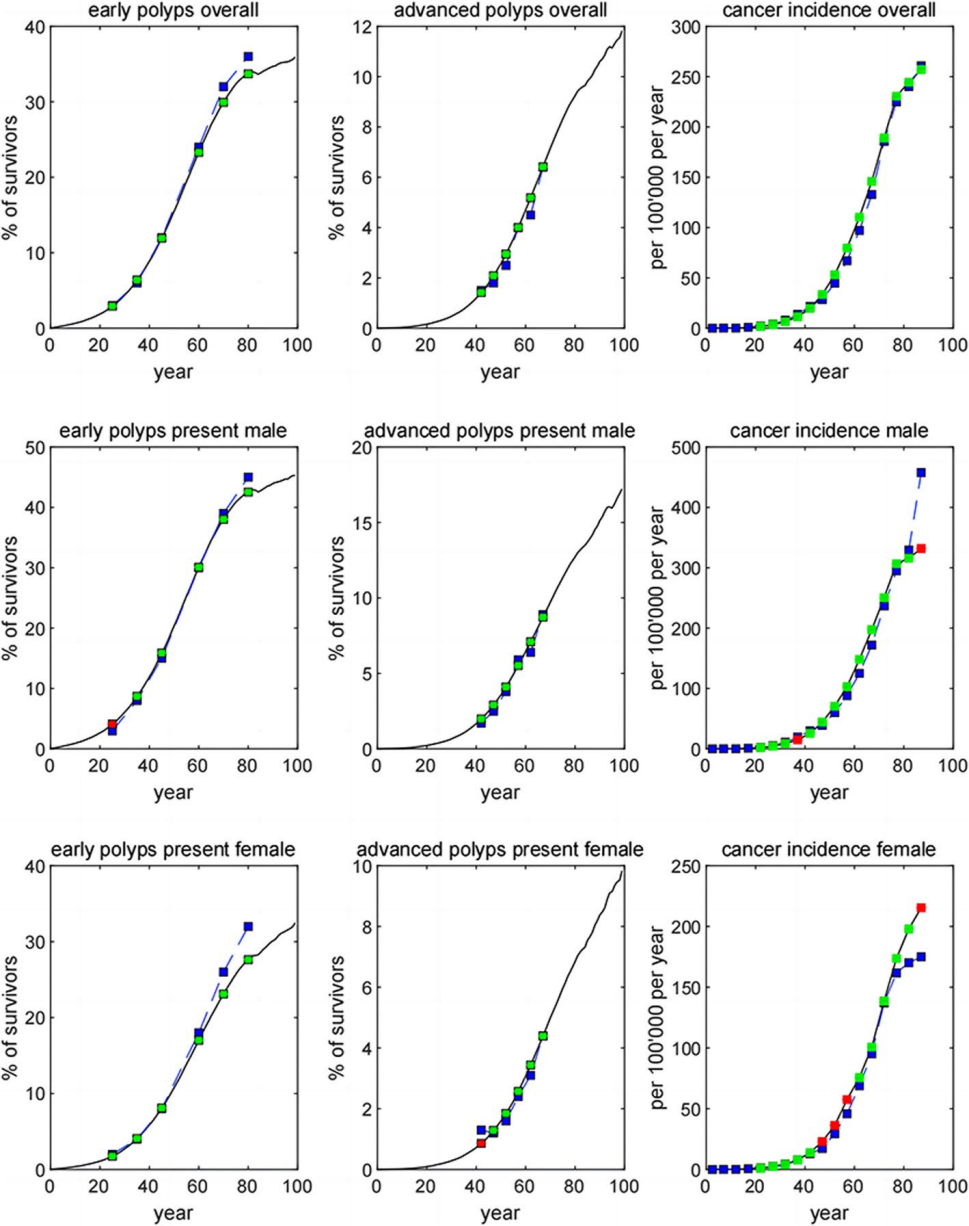
CMOST Calibrated by Chinese Benchmarks. Benchmarks were indicated by blue squares and a blue dashed line; results of CMOST simulation were shown as a black line and green-squares when within 20% of benchmarks, and as red-squares otherwise

Using CMOST, we simulated 1 million people without migration employing a common random number technique, from birth to death (or to maximum age of 100 years) with a 1:1 male-to-female ratio. The population size decreased with age due to nature death and cancer mortality. As a migrant city, residents in Shenzhen are from all over China, thus we utilized the China life table for 2020 to replace the US life table in the CMOST model; then we recalibrated the CMOST13 (a median dwell time of the three CMOST variants) according to the Chinese benchmarks before the pilot CRC screening in Shenzhen, including prevalence of advanced adenomas from 2012 to 2015 [20], incidence of CRC in year of 2019 [21], location of CRC in the rectum from 2002 to 2006 [22] and mortality of CRC in year of 2020 [23] (See supplemental file 1). The details of calibration were showed in supplemental file 2 and calibrated results were illustrated in Fig. 1. Except for a slightly higher CRC incidence among female aged 45–59 years, the majority of calibrated results fitted well with the benchmarks, including prevalence of early adenomas, prevalence of advanced adenomas and incidence of cancer among all groups of individuals.

### Screening strategies

We assessed three categories of screening tests, namely National Colorectal Polyp Care score (NCPCS) [24], FIT [25], and risk-stratification model (RS model) [24] (See Table 1). The NCPCS, a questionnaire-based risk assessment tool, calculates an individual risk score based on age, sex, smoking status and other characteristics. If the risk score exceeds a predefined threshold, a positive result is recorded. FIT is with a cutoff of 100 ng hemoglobin /mL. RS model which combines NCPCS and FIT, gets a positive result if at least one of them is positive. Individuals with a positive initial screening result underwent a diagnostic colonoscopy and surveillance colonoscopies if an adenoma or cancer was detected.

**Table 1:** Characteristics of different screening tests

Strategy	Model characteristics	Test characteristics	Value (%)	Source
**Colonoscopy**				
		Sensitivity for adenomas <=5 mm	65.0–75.0	Original set in the CMOST
		Sensitivity for adenomas 6 to 9 mm	81.0–87.0	
		Sensitivity for adenomas > = 1 cm	95.0	
		Sensitivity for CRC	95.0–100.0	
		Specificity	100.0	
**FIT**				
	Qualitative FIT for hemoglobin- Pupu Tube (New Horizon Health Technology, China), with the threshold of 100 ng hemoglobin /mL buffer specified by the manufacturer	Sensitivity for adenomas <=5 mm	0	Lu et al., 2021 [25]
		Sensitivity for adenomas 6 to 9 mm	12.9	Lu et al., 2021 [25]
		Sensitivity for adenomas > = 1 cm	33.9	Lu et al., 2021 [25]
		Sensitivity for CRC	83.3	Lu et al., 2021 [25]
		Specificity	90.2	Lu et al., 2021 [25]
		Participation rates	94.0	Chen et al., 2020 [26]
		Compliance to colonoscopy	76.3	Chen et al., 2020 [26]
**NCPCS**				
	Sex (male: 2; female: 0), Age (< 35: 0; 35–44: 7; 45–49: 9; 50–59: 11; ≥60: 13), BMI (< 24 kg/m^2^: 0; ≥24 kg/m^2^: 1), Smoking (current/past: 2; no: 0), Drinking (current: 2; no: 0), Diabetes (yes: 3; no: 0), FDR of CRC (yes: 2; no: 0), History of previous negative colonoscopy (PNC, yes: 0; no: 3). Intermediate/high risk criteria: Score ≥ 15.	Sensitivity for adenomas <=5 mm	NA	
		Sensitivity for adenomas 6 to 9 mm	68.7	Zhao et al., 2022 [24]
		Sensitivity for adenomas > = 1 cm	77.5	Zhao et al., 2022 [24]
		Sensitivity for CRC	78.7	Zhao et al., 2022 [24]
		Specificity	54.5	Zhao et al., 2022 [24]
		Participation rates	99.7	Chen et al., 2020 [26]
		Compliance to colonoscopy	49.2	Chen et al., 2020 [26]
**RS model**				
	Intermediate/high risk for NCPCS or FIT positive	Sensitivity for adenomas <=5 mm	NA	
		Sensitivity for adenomas 6 to 9 mm	70.9	Zhao et al., 2022 [24]
		Sensitivity for adenomas > = 10 mm	81.2	Zhao et al., 2022 [24]
		Sensitivity for CRC	93.6	Zhao et al., 2022 [24]
		Specificity	52.0	Zhao et al., 2022 [24]
		Participation rates	93.7	Chen et al., 2020 [26]
		Compliance to colonoscopy	77.0	Chen et al., 2020 [26]

*CRC* Colorectal cancer, *FIT* fecal immunochemical test, *NCPCS* National Colorectal Polyp Care score, *RS* Risk-stratification

NA was input as “0” in CMOST

The NCPCS, FIT and RS model have different sensitivity, specificity and participation assumptions (See Table 1). In CMOST, individuals with a false positive initial screening test do not continue screening till 5 years after their last colonoscopy, and other individuals are set to skip screening tests at random based on the rate of participation. We assessed several screening start-age (40, 45, 50 years), stop-age (70, 75, 80 years), and intervals (annual, biennial, triennial) for each strategy, resulting in a total of 81 screening strategies.

### Outcomes

Compared with no screening, the outcomes evaluated in this study included the benefits, burden, and harms of CRC screening. The benefits were assessed by life-years gained (LYG), CRC lifetime incidence and mortality reduction per 1000 individuals. The burden was quantified by number of lifetime colonoscopies per 1000 individuals, including both diagnostic and surveillance colonoscopies, and harms were measured by deaths and life-years lost per 1000 individuals due to colonoscopy.

### Data analysis

The model outputs were analyzed in R version 4.2.2. The efficiency of CRC screening across strategies was assessed by number of colonoscopies (input) VS LYG (output), using Data Envelopment Analysis (DEA) with a variable return-to-scale model (implemented via the R package “Benchmarking”). DEA is a non-parametric method for decision making, which involves the application of linear programming technique to estimate the performance. The core idea of DEA is to assess the efficiency by comparing the input(s) and output(s) of the decision-making units (DMUs), thus to determine a frontier of efficient DMUs that envelops all inefficient DMUs. Variable return-to-scale is one of the models for DEA to get the efficient frontier [27].

Within each of the three categories, the efficient frontier is the set of screening strategies that offer the highest LYG for a defined number of colonoscopies or require the fewest colonoscopies for a given number of LYG. And efficient strategies are those located on the efficient frontier. We compared these efficient strategies by the efficiency ratio, representing the additional colonoscopies per additional LYG compared with the default strategy (which required the fewest colonoscopies and provided the fewest LYG). We also calculated efficiency ratios for near-efficient strategies, which fell below the frontier but had LYG > 98% of the efficient frontier [28]. Hereafter in this study, the term “efficient” referred to both efficient and near-efficient strategies.

As burden of coloscopies was the critical medical resource input, we compared efficient strategies across different categories by assessing additional coloscopies per LYG (with the default being no screening; lower additional coloscopies per LYG indicates higher efficiency).

### Sensitivity analysis

Previous study has showed substantial impact of screening compliance on outcomes [19]. In this modelling study, the participation rates were derived from a multicenter randomized controlled trial involving 19,582 individuals [26] (see Table 1). Nevertheless, participation rates in mass population screening could be lower than those observed in experimental studies with relatively small populations. Therefore, we conducted sensitivity analyses with lower participation rates for FIT (42.0%) [29], NCPCS (15.4%) and RS model (14.0%) [30], as well as lower compliance rates for diagnostic colonoscopy following initial screening with FIT (64.8%), NCPCS (33.5%) and RS model (38.9%) [31].

As surveillance colonoscopy is not considered in Chinese guidelines [32], we also conducted additional analyses of screening strategies without surveillance colonoscopies for adenoma and cancer.

## Results

### Benefits, burden, and harms of screening

Without screening, the CRC lifetime incidence was 46 per 1000 individuals, with a CRC lifetime mortality of 17. Compared with no screening, the screening strategies reduced CRC lifetime incidence by 14–27 cases (30.9–59.0%) and lifetime mortality by 7–12 deaths (41.5–71.3%), while yielded 83–155 LYG. Annual RS model screening from age 40 to 80 years was the most effective strategy in reducing CRC incidence, while annual FIT screening with the same age range reduced the most mortality and yielded the most LYG.

Across all screening strategies, the lowest number of colonoscopies was required for triennial FIT screening from age 50 to 70 years (920 colonoscopies), while the highest number of colonoscopies was required for annual RS model screening from age 40 to 80 years (5901 colonoscopies). Within each category of screening tests, the estimated number of lifetime colonoscopies increased when screening was initiated at an earlier age, stopped at an older age, and conducted at shorter intervals (See supplemental file 3).

Despite the high number of colonoscopies, the incidences of harms were low, with at most of 0.20 deaths and 4.73 years lost per 1000 individuals due to colonoscopy for annual RS model screening from age 40 to 80 years.

### Efficiency analysis

**Fig. 2:**
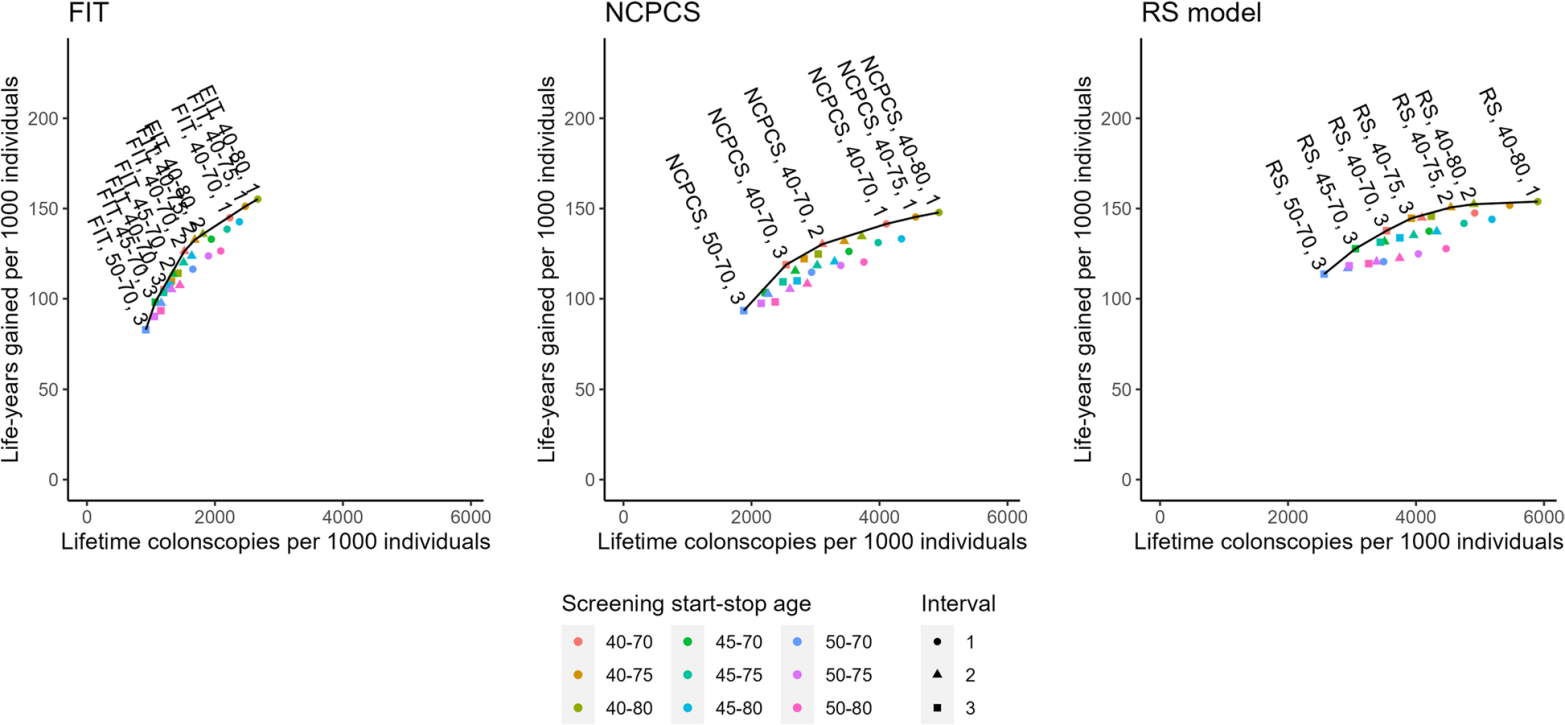
Lifetime colonoscopies and life-years gained for difference screening strategies, efficient frontier, and efficient strategies. Efficient frontier was showed as black line. FIT: Fecal Immunochemical Test, NCPCS: National Colorectal Polyp Care score, RS: Risk-stratification

Figure 2 illustrated the estimated lifetime number of colonoscopies and LYG per 1000 individuals for each category of screening tests, as well as the efficient frontier and efficient strategies. Among the total of 81 screening strategies evaluated, 23 strategies were estimated efficient (See Table 2).

**Table 2:** Outcomes per 1000 individuals for efficient screening strategies within the CMOST model

Strategy	COLs	Non-COL tests	Reduced CRC cases	Reduced CRC deaths	LYG	Patients died of COLs	Years lost due to COLs	Efficiency ratio (ΔCOL/ΔLYG)	Ratio of additional COLs to LYG
**FIT**									
FIT, 50–70, 3	920	6013	14	7	83	0.04	0.67	Default	10.5
FIT, 45–70, 3	1068	7542	16	8	98	0.03	0.71	9.6	10.4
FIT, 40–70, 3^a^	1202	9064	17	8	105	0.05	1.05	12.7	11.0
FIT, 45–70, 2^a^	1344	10,342	19	9	114	0.06	1.27	13.7	11.4
FIT, 40–70, 2	1522	12,444	20	9	126	0.05	1.09	13.8	11.7
FIT, 40–75, 2	1685	13,954	21	10	133	0.06	1.22	15.4	12.3
FIT, 40–80, 2	1812	15,196	21	11	136	0.06	1.25	16.8	13.0
FIT, 40–70, 1^a^	2232	20,461	23	11	145	0.09	2.26	21.1	15.1
FIT, 40–75, 1	2476	22,938	24	12	151	0.10	2.25	22.7	16.1
FIT, 40–80, 1	2670	24,963	24	12	155	0.10	2.23	24.2	16.9
**NCPCS**									
NCPCS, 50–70, 3	1880	5585	19	8	93	0.07	1.32	Default	19.6
NCPCS, 40–70, 3	2542	8294	22	9	119	0.1	2.35	26.0	21.0
NCPCS, 40–70, 2	3107	10,553	24	10	130	0.12	2.66	33.3	23.5
NCPCS, 40–70, 1	4107	14,715	25	11	141	0.13	3.23	46.3	28.7
NCPCS, 40–75, 1	4565	16,440	26	12	145	0.15	3.32	51.7	31.1
NCPCS, 40–80, 1	4930	17,855	27	12	148	0.16	3.41	56.0	33.0
**RS model**									
RS model, 50–70, 3	2564	4992	22	10	114	0.08	1.47	Default	22.1
RS model, 45–70, 3	3054	6220	24	10	128	0.12	2.53	34.7	23.5
RS model, 40–70, 3	3539	7475	24	10	138	0.11	2.60	40.6	25.3
RS model, 40–75, 3	3928	8346	26	11	145	0.12	2.83	44.1	26.8
RS model, 40–75, 2	4543	9899	26	12	151	0.14	3.50	53.5	29.8
RS model, 40–80, 2	4903	10,748	27	12	152	0.14	3.39	60.4	31.9
RS model, 40–80, 1	5901	13,325	27	12	154	0.20	4.73	83.1	38.0

*COL* Colonoscopy, *CRC* Colorectal cancer, *LYG* Life-years gained, *FIT* Fecal immunochemical test, *RS* Risk-stratification, *NCPCS* National Colorectal Polyp Care score; *Default*, the one with the least LYG and required least colonoscopy, *ΔCOL*, incremental number of colonoscopies compared with the default strategy, *ΔLYG*, incremental number of LYG compared with the default strategy. Additional COLs were the difference between efficient strategy and no screening

^a^ Near-efficient strategies

Most of the efficient screening strategies were those starting at age 40 (17 out of 23 strategies) and stopping at age 70 years (13 out of 23 strategies). For FIT, efficient strategies included annual and biennial screening starting at age 40 and stopping at age 70, 75 and 80 years, as well as triennial screening commencing at age 40, 45, or 50 and ending at age 70 years. Of the six efficient screening strategies based on NCPCS, three began at age 40 and ceased at age 70 years. Similarly, five RS model screening strategies started at age 40 years, with three stopping at age 70 years.

Among the different categories of efficient screening strategies, annual screening was predominant for NCPCS, biennial for the FIT, and triennial for the RS models. Generally, the efficiency ratios increased with shorter intervals. For instance, with FIT screening from ages 40 to 70, the efficiency ratio was 12.7 for triennial screening, rising to 13.8 and 21.1 for biennial and annual screening, respectively. Similarly, for NCPCS screening from age 40 to 70, the efficiency ratios for triennial, biennial and annual screening were 26.0, 33.3 and 46.3, respectively.

Among all 23 efficient strategies, when screening at the same start-to-stop age and interval, the additional colonoscopies per LYG increased progressively for FIT, NCPCS and RS model. For example, with triennial screening from age 40 to 70 years, FIT required 1202 colonoscopies and yielded 105 LYG, resulting in 11.0 additional colonoscopies per LYG; NCPCS required 2542 colonoscopies (increased 111.5%), but yielded only 119 LYG (an increasing of 13.3%), with 21.0 additional colonoscopies per LYG; while RS model required 3539 colonoscopies (a 39.2% increasing compared with NCPCS), yielding 138 LYG (a 16.0% increasing over NCPCS), and resulting in 25.3 additional colonoscopies per LYG.

### Sensitivity analysis

Compared with the screening strategies in the main analysis (characterized by high participation rates and with surveillance colonoscopy), screening strategies with lower participation rates necessitated 0 to 77.5% fewer colonoscopies; however, these strategies leaded to 0 to 73.7% more CRC cases and 25.5 to 65.2% fewer LYG. Similarly, screening strategies without surveillance colonoscopy required 0 to 40.8% fewer colonoscopies, resulting in 0 to 20.8% more CRC cases and 0 to 16.1% fewer LYG (See supplemental file 3).

Lower participation rates during initial screening and lower compliance rates for diagnostic colonoscopy did not significantly affect the findings. The majority of efficient strategies still started at age 40 years (13 out of 21 strategies) and stopped at age 70 years (14 out of 21 strategies). And when screening at the same start-to-stop ages and intervals, the additional colonoscopies per LYG increased progressively for FIT, NCPCS and RS model. Different from outcomes observed in the analysis with high participation rates, annual screening was the most efficient interval for the FIT, while no interval was predominated for the RS models in this sensitivity analysis. Moreover, annual RS model screening commenced at age 40 and stopped at age 70 or 75 years was proved efficient (See Table 3).

**Table 3:** Outcomes per 1000 individuals for efficient screening strategies within the CMOST (low participation and compliance rates for screening)

Strategy	COLs	Non-COL tests	Reduced CRC cases	Reduced CRC deaths	LYG	Patients died of COLs	Years lost due to COLs	Efficiency ratio (ΔCOL/ΔLYG)	Ratio of additional COLs to LYG
**FIT**									
FIT, 50–70, 3	653	4306	10	5	59	0.03	0.45	Default	10.3
FIT, 40–70, 3	829	6432	13	6	77	0.03	0.58	9.7	10.1
FIT, 40–70, 2	969	7996	14	7	89	0.04	0.76	10.3	10.3
FIT, 40–75, 2^a^	1071	8993	15	8	96	0.04	0.79	11.4	10.7
FIT, 40–70, 1	1198	10,697	17	8	107	0.04	0.95	11.4	10.8
FIT, 40–75, 1	1327	12,029	18	9	111	0.04	0.83	12.9	11.5
FIT, 40–80, 1	1428	13,121	18	9	114	0.04	0.95	14.1	12.1
**NCPCS**									
NCPCS, 50–70, 3	680	2178	8	4	36	0.01	0.27	Default	17.6
NCPCS, 50–70, 2^a^	726	2384	9	4	39	0.02	0.39	16.1	17.5
NCPCS, 45–70, 2	841	2983	10	4	48	0.03	0.52	12.7	16.4
NCPCS, 40–70, 1	1034	3995	12	5	58	0.03	0.54	16.1	17.1
NCPCS, 40–75, 1	1144	4498	13	5	59	0.04	0.57	20.0	18.6
NCPCS, 40–80, 1	1230	4912	13	6	60	0.04	0.69	22.8	19.7
**RS Model**									
RS model, 50–70, 3	741	2007	9	4	42	0.03	0.53	Default	16.7
RS model, 50–70, 2^a^	785	2174	9	4	45	0.03	0.68	12.6	16.3
RS model, 45–70, 3	859	2508	10	5	51	0.03	0.49	12.3	15.8
RS model, 45–70, 2^a^	913	2721	11	5	54	0.04	0.70	13.7	16.0
RS model, 40–70, 3	973	3018	11	5	59	0.04	1.03	13.3	15.7
RS model, 40–70, 1	1114	3596	13	5	64	0.05	1.26	16.8	16.7
RS model, 40–75, 1	1235	4048	13	6	66	0.06	1.30	19.9	17.9
RS model, 40–80, 1	1329	4418	14	6	66	0.05	1.24	23.7	19.3

*COL* Colonoscopy, *CRC* Colorectal cancer, *LYG* Life-years gained, *FIT* Fecal immunochemical test, *RS* Risk-stratification, *NCPCS* National Colorectal Polyp Care score; *Default*, the one with the least LYG and required least colonoscopy, *ΔCOL*, incremental number of colonoscopies compared with the default strategy, *ΔLYG*, incremental number of LYG compared with the default strategy. Additional COLs were the difference between efficient strategy and no screening

^a^ Near-efficient strategies

In addition, our results were robust when surveillance colonoscopy was not considered. Most of efficient screening strategies were started at age 40 years (21 out of 29 strategies) and stopped at age 70 years (11 out of 29 strategies). However, triennial screening was predominant for the FIT and NCPCS, and more NCPCS screening strategies demonstrated efficient than the main analysis (See Table 4).

**Table 4:** Outcomes per 1000 individuals for efficient screening strategies within the CMOST model (no surveillance colonoscopy)

Strategy	COLs	Non-COL tests	Reduced CRC cases	Reduced CRC deaths	LYG	Patients died of COLs	Years lost due to COLs	Efficiency ratio (ΔCOL/ΔLYG)	Ratio of additional COLs to LYG
**FIT**									
FIT, 50–70, 3	545	6228	11	6	76	0.02	0.48	Default	6.5
FIT, 45–70, 3^a^	663	7782	12	6	85	0.03	0.68	12.6	7.2
FIT, 45–75, 3^a^	765	9010	13	7	92	0.03	0.68	13.2	7.7
FIT, 40–75, 3	877	10,496	14	8	103	0.03	0.74	12.3	8.1
FIT, 40–70, 2	1053	12,896	15	8	117	0.04	0.97	12.4	8.6
FIT, 40–75, 2	1188	14,565	17	9	125	0.04	1.02	13.1	9.1
FIT, 40–80, 2	1304	15,961	18	10	129	0.05	1.11	14.2	9.7
FIT, 40–75, 1	1926	24,233	21	11	147	0.07	1.48	19.3	12.7
FIT, 40–80, 1	2114	26,563	22	12	152	0.08	1.57	20.5	13.6
**NCPCS**									
NCPCS, 50–70, 3	1415	5938	15	7	78	0.05	1.16	Default	17.4
NCPCS, 45–70, 3^a^	1725	7307	17	7	91	0.06	1.39	24.2	18.4
NCPCS, 40–70, 3	2040	8718	18	8	104	0.07	1.71	24.1	19.1
NCPCS, 40–75, 3^a^	2303	9843	20	9	110	0.08	1.74	28.1	20.5
NCPCS, 40–80, 3^a^	2521	10,775	21	9	114	0.09	1.85	30.7	21.6
NCPCS, 40–70, 2	2594	11,166	20	8	116	0.08	2.33	31.0	21.9
NCPCS, 40–75, 2	2927	12,595	21	9	122	0.1	2.46	34.3	23.5
NCPCS, 40–80, 2^a^	3206	13,791	23	10	126	0.11	2.65	37.7	25.1
NCPCS, 40–70, 1^a^	3615	15,712	22	9	130	0.11	2.98	42.2	27.3
NCPCS, 40–75, 1^a^	4076	17,715	24	10	137	0.13	3.37	45.5	29.4
NCPCS, 40–80, 1	4463	19,391	25	11	142	0.15	3.38	48.1	31.1
**RS model**									
RS model, 50–70, 3	2083	5386	19	8	104	0.09	1.98	Default	19.6
RS model, 45–70, 3	2563	6670	20	9	119	0.09	2.25	30.5	21.0
RS model, 45–75, 3^a^	2947	7675	22	10	126	0.10	2.50	39.7	23.1
RS model, 40–70, 3	3036	7956	21	9	128	0.10	2.69	39.1	23.3
RS model, 40–75, 3	3420	8961	23	10	135	0.11	2.81	43.2	25.0
RS model, 40–80, 3	3743	9804	24	11	140	0.13	2.86	46.2	26.5
RS model, 40–75, 2	4056	10,675	24	11	143	0.16	3.85	49.8	28.0
RS model, 40–80, 2	4439	11,680	25	12	148	0.17	3.90	53.5	29.7
RS model, 40–80, 1	5509	14,572	26	12	152	0.18	4.31	71.1	35.9

*COL* Colonoscopy, *CRC* Colorectal cancer, *LYG* Life-years gained, *FIT* Fecal immunochemical test, *RS* Risk-stratification, *NCPCS* National Colorectal Polyp Care score; *Default*, the one with the least LYG and required least colonoscopy, *ΔCOL*, incremental number of colonoscopies compared with the default strategy, *ΔLYG*, incremental number of LYG compared with the default strategy. Additional COLs were the difference between efficient strategy and no screening

^a^ Near-efficient strategies

## Discussion

In this study, most types of screening strategies (FIT, questionnaire-based risk assessment tool, or both) current used in China were modelled. We identified efficient organized CRC screening strategies for Shenzhen City. In the background of incomplete participation rates of CRC screening and diagnostic colonoscopy, whether surveillance colonoscopy was considered or not, the efficient screening strategies generally began at age of 40 years and stopped at age of 70. When performed with the same start-to-stop age and interval, the FIT screening strategy required the fewest colonoscopies and yield relevant LYG, followed by NCPCS and RS model screening. The microsimulation study provides valuable insights into the efficient CRC screening strategies in Shenzhen, and could inform the development of organized CRC screening programs.

To the best of our knowledge, this is the first microsimulation modeling study in China that included two CRC developing pathways and adopted real-world participation rates to evaluate CRC screening strategy. Previous modelling studies on CRC screening in China, such as the MISCAN-Colon modelling studies in Shanghai [16, 17], only considered the adenoma-carcinoma pathway. Additional, Lu et al. established the MIMIC-CRC model and applied it to simulate several screening protocols involving FIT and colonoscopy; however, it’s important to notice that the MIMIC-CRC model functions as a Markov model and cannot simulate the dynamic changes in individual characters as a microsimulation model does [33]. Furthermore, Zhou et al. conducted a cost-effectiveness analysis of different CRC screening strategies in Guangzhou [34]. After comparing four annual screening strategies for individuals aged 50–74 years, they suggested the double FIT was the most cost-effective one; but also, the analysis was based on a Markov model.

In this modeling study, under conditions of relatively high participation rates, we observed that longer screening intervals among efficient strategies were associated with more targeted age ranges. For instance, in the main analysis, annual FIT screening starting at age 40 and ending at 75 or 80 years demonstrated efficiency. While for triennial FIT screening, efficient strategies involved initiating screening at age 45 or 50 and concluding at 70 years. The finding aligns closely with a modeling study conducted in Shanghai [16], which identified annual FIT screening (with cut-off of 10 μg Hb/g faeces taking one sample, FIT-1-10) starting at either age 45 or 50 and concluding at age 80 years as an efficient approach. And for triennial FIT-1-10, it recommended initiation at age 50 and conclusion at age 70 years.

Our efficient FIT screening strategies closely resemble those identified in the updated modeling study for the US Preventive Services Task Force [10], although with starting ages 5 years earlier. While the majority of efficient strategies identified in the US study initiated screening at age 45, ours predominantly commenced at age 40 years. The discrepancy between the US study and our model may be attributed to the difference in simulated age ranges for screening, i.e., the US study did not model screening strategies with initiation at age 40 (starting at 45, 50 and 55 years, and stopping at 70, 75, 80, and 85 years), whereas our model did not simulate screening strategies with cessation age of 85 years.

Young-onset CRC or “early-onset” CRC arises globally in recent years, with an even higher increase of CRC incidence among adults ages 30–39 years [35, 36]. According to the microsimulation modelling results, 2021 US Preventive Service Task Force guideline suggested routine screening from ages 45 to 75 years, hence lowering the age to begin CRC screening from 50 to 45 years [10]. In China, there is no unified standard for the starting age of CRC screening for average-risk individuals in the majority of guidelines [6]. The surveillance results of CRC in Shenzhen show an increase of CRC incidence from age 40 years, with a significant upward trend starting at age 45 [8]. Currently, the CRC screening pilot program in Shenzhen starts at age of 45 years. In this simulation study, it showed that there were only three efficient strategies starting at age 45 years, and efficiency ratios (additional colonoscopies per additional LYG) slightly increased when lowing the age to begin screening from 45 to 40 years.

The choice of screening type depends on medical resources. In the China Guidelines for Screening, Early Diagnosis and Treatment of Colorectal Cancer (2020, Beijing), questionnaire-based risk assessment tools are suggested for CRC risk assessments, identifying high-risk populations for CRC screening using FIT, sigmoidoscopy or colonoscopy, thus to save health resources [32]. In this simulation study, the RS model which combined NCPCS and FIT, required highest additional colonoscopies per LYG, while FIT required the least, and NCPCS alone yielded comparable LYG to FIT with given additional colonoscopies. Recently, FIT has shown comparative high specificity and sensitivity among noninvasive CRC screening tests, and is covered by all screening guidelines. It appears that FIT and NCPCS alone, rather than the RS model, could be used as efficient CRC screening strategies within insufficient colonoscopy resources.

There are several other widely used questionnaire-based risk assessment tools in China, such as Asia Pacific Colorectal Screening score (APCS) [37], Modified APCS [38], high risk factors questionnaire (HRFQ) [39, 40], individual cancer risk assessment questionnaire based on the theory of Harvard Cancer Risk Index [41], and a logistical prediction model [42]. Among these models, the HRFQ demonstrates comparatively low sensitivity for CRC detection (24.5%) [39]. The logistical prediction model exhibits a sensitivity of 82.8% and specificity of 50.8% for CRC detection [42], which is close to that of NCPCS. Absent age- and gender-specific sensitivities and specificities, there is a potential bias for APCS and Modified APCS during CMOST simulations, such as the overestimation of APCS sensitivity for females aged 40–49, leading to more diagnostic colonoscopies. Additionally, to our knowledge, other mentioned questionnaire-based risk assessment tools have not been evaluated for their sensitivity and specificity in detecting early adenomas and advanced adenomas, which makes them incompatible with the CMOST model.

As the effectiveness of a screening strategy relies significantly on participation, the optimal screening strategy proposed with 100% adherence may not be feasible in practice. Acknowledging the challenge of attaining full adherence in real-world scenarios, unlike conventional methodologies assuming complete participation, we conducted simulations of screening programs using observed participation rates from randomized trials, which provide a more realistic portrayal of achievable adherence levels in real-world settings. Wang et al. similarly utilized real-world participation rates to simulated the effects of CRC screening in Shanghai [17].

Initial screening interval significantly influences screening participation rates, and conversely, diverse participation rates necessitate distinct initial screening intervals. A system-review and meta-analysis showed that initial tests were the most modifiable factor for adherence to diagnostic colonoscopy, with gFOBT/FIT showing the highest adherence to colonoscopy amongst the noninvasive initial screening [43]. Additionally, several studies examined participation rates and effects of repeated CRC screening. Fisher et al. demonstrated a decline in adherence to repeat FIT tests over subsequent years, with adherence rates of 23.4% at year 2 and 10.6% at year 3 [44]. And van Roon et al. illustrated that triennial screening had higher participation rate for FIT than annual screening in the second round, and there was no impact on advanced neoplasia detection within intervals ranging from 1 to 3 years [45]. In this study, we observed biennial was predominated for FIT screening strategies when the participation rates were high, while annual screening was predominated in scenarios of low participation rates. Policy makers should select an appropriate initial screening interval based on the participation rate to achieve optimal screening effects, while also being awareness of adherence patterns. For example, if individuals participate to a screening test randomly, reducing the screening interval could enhance optimality. However, if certain individuals consistently avoid screening while others attend all screening rounds, decreasing screening interval may not yield significant benefits.

## Limitation

It is worth noting that the study has several limitations. Firstly, the values of sensitivity and specificity for NCPCS in this study are derived solely from a single literature source. Secondly, the model lacks the capability to simulate varying participation rates during repeated screening. Thirdly, the study does not incorporate quality-adjusted health outcomes, such as “Disability-adjusted life years”, as it is not available for the CMOST. Additionally, CMOST assumes that sessile-serrated polyps (SSPs) are never detected by colonoscopy screening for the Serrated-Cancer pathway, however, currently percents of SSPs are found in tandem colonoscopies [46]. Furthermore, NCPCS is inherently influenced by age in some certain, resulting in a potential underestimation of diagnostic colonoscopies among individuals aged 60 and above in CMOST, while overestimating them in younger individuals. This age-dependency does not only bias the number of colonoscopies but also affects other screening outcomes. The disparities in screening outcomes between NCPCS/RS strategies simulated in this study and real-world scenarios should be noticed.

Lastly, there is no clear way to interpret what is the limit on the efficiency ratios that emerge from efficiency analysis. When considering only the burden of colonoscopy, given a threshold of 20 for efficiency ratio within the FIT strategies for example, the optimal policy would favor triennial and biennial screening over annual screening. And with a threshold of 15, it should not be to extend screening to age 70. It is essential to note that the acceptability of efficiency strategies should be evaluated in the context of cost-effectiveness analysis, which encompassed economic costs for treatments, screening tests, side effects of colonoscopies and so on, and should be judged by incremental cost-effective ratios (ICERs). For instance, based on a willingness-to-pay threshold set at three times the Chinese gross domestic product per capita for one LYG, the Shanghai model study suggested the optimal efficient screening is the annual FIT-1-10 screening from ages 45 to 80 years [16]. However, to our knowledge, as Chinese economic costs for CRC treatments and side effects that fit the CMOST model is absent (requiring costs of initial, continuous and terminal treatment for types of CRC, and so on), we cannot calculate the costs and ICERs for efficient strategies.

Future research should address these limitations and explore the cost-effectiveness of various screening strategies.

## Strength

In this study, we used both Adenoma-Carcinoma and Serrated-Cancer pathways to simulate a comprehensive history of colorectal cancer. Secondly, we used participation rates from real world to evaluate screening strategies, which might be more accurate than empirical derivations. Overall, this study provides valuable insights into the efficient CRC screening strategies for Shenzhen and other similar regions. The identified efficient strategies can help policymakers and healthcare providers to develop and implement effective organized CRC screening programs that are tailored to the local population’s needs and resources.

## Conclusion

This study identifies efficient CRC screening strategies for the average-risk population in Shenzhen. Most efficient screening strategies indeed start at age 40, but the optimal starting age depends on the chosen willingness-to-pay threshold. Within insufficient colonoscopy resources, efficient FIT and NCPCS screening strategies might be CRC initial screening strategies.

### Supplementary Information

**Supplementary Material 1.**

**Supplementary Material 2.**

**Supplementary Material 3.**

## Data Availability

All data generated or analyzed during this study are included in this published article and its online supplementary files.

## Abbreviations

APCS: Asia pacific colorectal screening score
CMOST: Colon modeling open simulation tool
CMOST13: CMOST model with a dwell time of 13 years
CRC: Colorectal cancer
FIT: Fecal immunochemical test
gFOBT: Guaiac fecal occult blood test
HRFQ: High risk factors questionnaire
LYG: Life-years gained
NCPCS: National Colorectal Polyp Care score
RS model: Risk-stratification model
SSPs: Sessile-serrated polyps
VRS: Variable return-to-scale
ICERs: Incremental cost-effective ratios
