# Supplementary material for: Efficient organized colorectal cancer screening in Shenzhen: a microsimulation modelling study
Source: BMC Public Health. 2024 Mar 1;24:655. doi: 10.1186/s12889-024-18201-w (PMC10905924; doi:10.1186/s12889-024-18201-w)
Supplement: Supplementary file 1 — Supplementary Material 1. [file 12889_2024_18201_MOESM1_ESM.docx]

**Benchmark 1:** Advanced adenoma prevalence (percent of population with ≥1 advanced adenoma) ^1^

| **Age (years)** | 42 | 47 | 52 | 57 | 62 | 67 |
| --- | --- | --- | --- | --- | --- | --- |
| **Advanced adenomas, overall (%)** | 1.5 | 1.8 | 2.5 | 4.0 | 4.5 | 6.4 |
| **Advanced adenomas, male(%)** | 1.7 | 2.5 | 3.8 | 5.9 | 6.4 | 8.9 |
| **Advanced adenoma, female(%)** | 1.1 | 1.2 | 1.6 | 2.4 | 3.0 | 4.4 |

**Benchmark 2:** Cancer incidence (per 100,000 per year)^2^

| Age | Overall | Males | Females |
| --- | --- | --- | --- |
| 1-4 years | 0.00 | 0.00 | 0.00 |
| 5-9 years | 0.11 | 0.12 | 0.10 |
| 10-14 years | 0.12 | 0.12 | 0.12 |
| 15-19 years | 0.89 | 1.10 | 0.65 |
| 20-24 years | 2.14 | 2.76 | 1.47 |
| 25-29 years | 3.95 | 5.29 | 2.56 |
| 30-34 years | 7.86 | 11.12 | 4.51 |
| 35-39 years | 13.86 | 19.61 | 7.88 |
| 40-44 years | 21.62 | 29.95 | 12.94 |
| 45-49 years | 28.42 | 39.15 | 17.27 |
| 50-54 years | 44.83 | 59.97 | 29.55 |
| 55-59 years | 67.03 | 88.02 | 45.85 |
| 60-64 years | 97.11 | 125.10 | 68.86 |
| 65-69 years | 132.84 | 171.92 | 95.16 |
| 70-74 years | 185.64 | 236.69 | 137.09 |
| 75-79 years | 224.81 | 294.84 | 161.79 |
| 80-84 years | 240.12 | 329.43 | 170.16 |
| 85+years | 260.77 | 457.47 | 174.97 |

**Benchmark 3:** Percentage of rectal carcinoma of all carcinoma^3^

| Rectal carcinoma percentage (age group) | 41-45 | 51-55 | 61-65 | 71-75 |
| --- | --- | --- | --- | --- |
| Percentage male | 47.2 | 51 | 48.5 | 42.4 |
| Percentage female | 46.7 | 45.7 | 39.8 | 36.7 |

**Benchmark 4:** Colorectal cancer mortality^4^

| Age (years) | overall | males | females |
| --- | --- | --- | --- |
| 1-4 years | 0 | 0 | 0 |
| 5-9 years | 0 | 0 | 0 |
| 10-14 years | 0.01 | 0 | 0.01 |
| 15-19 years | 0.11 | 0.13 | 0.08 |
| 20-24 years | 0.24 | 0.23 | 0.26 |
| 25-29 years | 0.45 | 0.54 | 0.35 |
| 30-34 years | 0.99 | 1.13 | 0.86 |
| 35-39 years | 1.42 | 1.44 | 1.4 |
| 40-44 years | 2.75 | 3.13 | 2.35 |
| 45-49 years | 4.3 | 5.11 | 3.47 |
| 50-54 years | 7.48 | 9.1 | 5.85 |
| 55-59 years | 12.72 | 16.37 | 9.07 |
| 60-64 years | 20.46 | 27 | 13.86 |
| 65-69 years | 34.38 | 44.8 | 24.36 |
| 70-74 years | 56 | 71.76 | 41.08 |
| 75-79 years | 85.27 | 108.15 | 65.18 |
| 80-84 years | 126.36 | 162.43 | 98.48 |
| 85+ years | 169.19 | 217.69 | 138.1 |

**The implemented surveillance structure in CMOST^5^:**

- **adenoma surveillance**: surveillance colonoscopies will be performed 5 years after detection of 1 or 2 early adenomas, 3 years after detection of an advanced adenomas, and 5 years henceforward.
- **cancer surveillance**: CRC surveillance colonoscopies will be performed 1 and 4 years after CRC detection and henceforward every 5 years.

**Reference:**

1. Chen H, Li N, Ren J, Feng X, Lyu Z, Wei L, et al. Participation and yield of a population-based colorectal cancer screening programme in China. Gut. 2019; 68(8):1450-1457.

2. Global Burden of Disease Study 2019 (GBD 2019) Results. Availble from: https://vizhub.healthdata.org/gbd-results/. Accessed on 2 July 2023.

3. Yangming G, Chunxiao W, Minlu Z, Peng P, Kai G, Pingping B, et al. [Colorectal cancer survival analysis in major areas in Shanghai China]. [China Oncology]. 2015; 25(7):497–504.

4. Cause of death surveillance data set in China. 2020. In*.* Beijing: China Science and Technology Press; 2021.

5. Prakash MK, Lang B, Heinrich H, Valli PV, Bauerfeind P, Sonnenberg A, et al. CMOST: an open-source framework for the microsimulation of colorectal cancer screening strategies. BMC Med Inform Decis Mak. 2017; 17(1):80.
