# Supplementary material for: Efficient organized colorectal cancer screening in Shenzhen: a microsimulation modelling study
Source: BMC Public Health. 2024 Mar 1;24:655. doi: 10.1186/s12889-024-18201-w (PMC10905924; doi:10.1186/s12889-024-18201-w)

**Details of CMOST calibration process**

In general, we replaced the US life table with the Chinese life table and made adjustments to specific benchmarks in the default benchmarks file of CMOST. These benchmarks include the prevalence of advanced adenomas, incidence of colorectal cancer (CRC), location of CRC in the rectum, and mortality of CRC. We employed the automated calibration steps outlined in the CMOST manual, progressing through steps 1 to 3 sequentially, to recalibrate these benchmarks. The following details provide a comprehensive overview of the automated calibration steps.

**1. Step 1 (Calibration for early adenomas prevalence and distribution)**

We utilized the original benchmarks established by CMOST, adjusted the "Auto_Calibration_Step_1.m" file, and executed the calibration process as outlined in the CMOST manual.

-
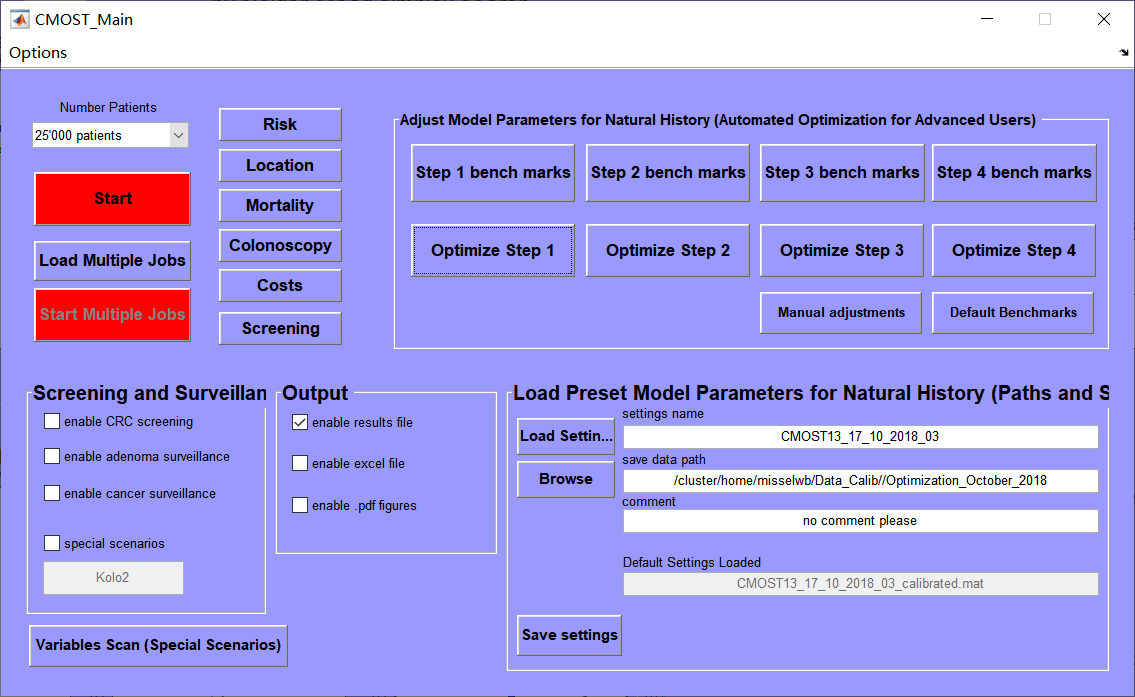
First, we loaded the "CMOST13_17_10_2018_03_calibrated.mat" within the CMOST environment, configured a simulated population of 25,000, and deactivated the CRC screening, adenoma surveillance, and cancer surveillance flags.
- Second, we adjusted the file of “Auto_Calibration_Step_1.m”, in which we modified early adenomas parameters:


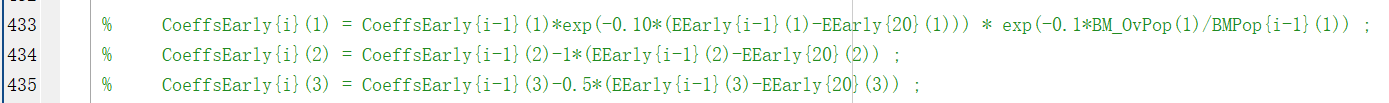


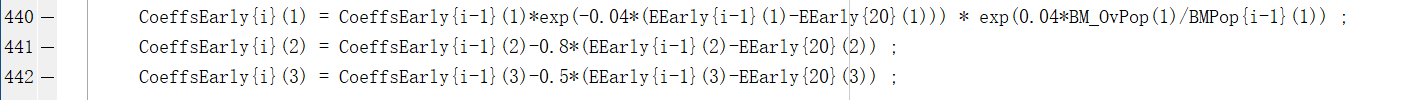
.

Note: The commented part is the original parameter.

- Third, the iterations were set 10 for first and 30 for second as suggested by manual.


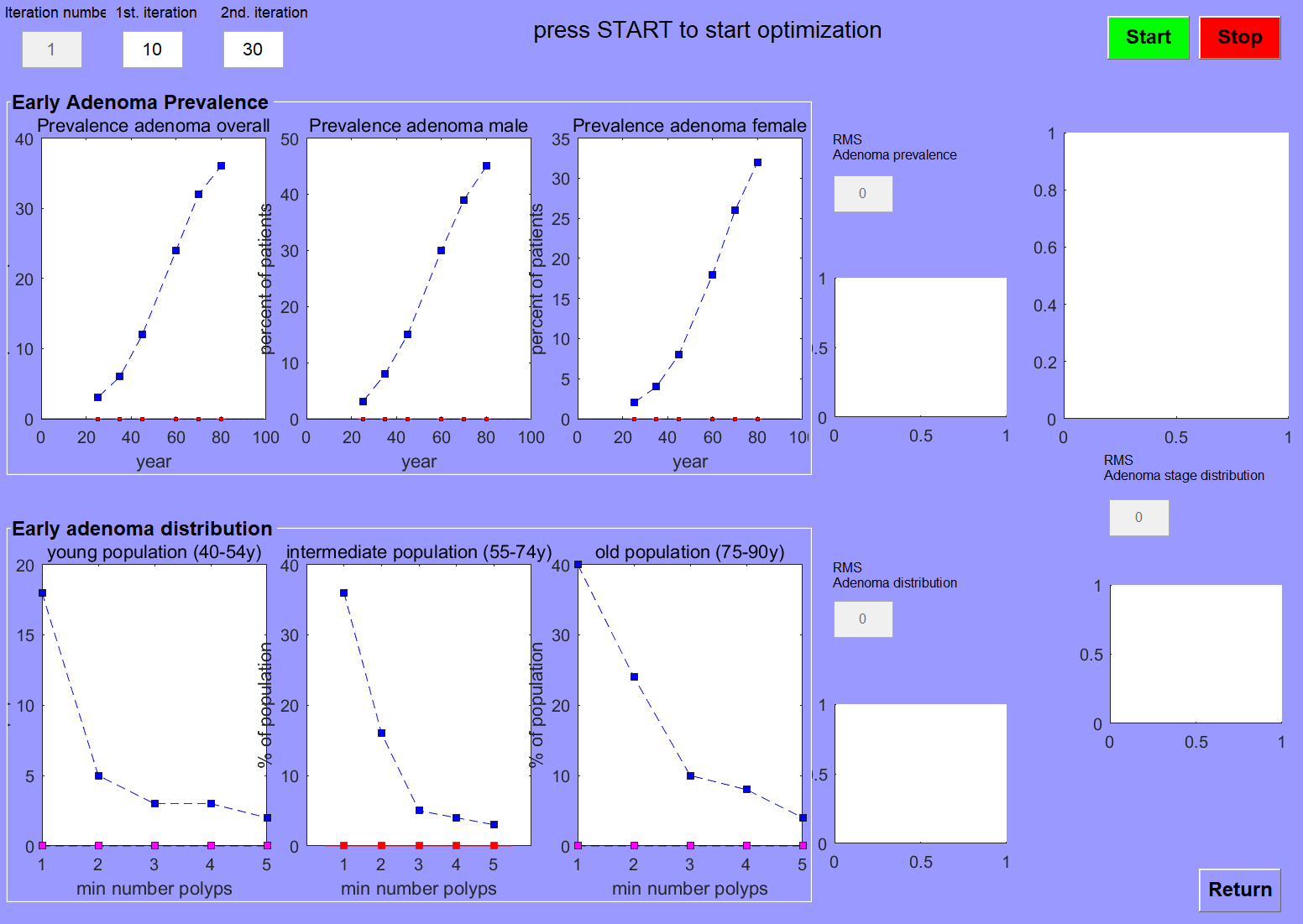


**2. Step 2 (Calibration for** **advanced adenomas prevalence and distribution)**

We utilized the prevalence data of advanced adenomas in China from 2012 to 2015 as our calibration targets. The calibration process was conducted following the instructions outlined in the CMOST manual and involved adjustments to the "Auto_Calibration_Step_2.m" file.

- First, we set a simulated population of 50,000, also disabled flags of CRC screening, adenomas surveillance and cancer surveillance.
- Second, we adjusted the file of “Auto_Calibration_Step_2.m”:

We adjusted the initial value during the nonlinear fitting for advanced adenomas prevalence curve：
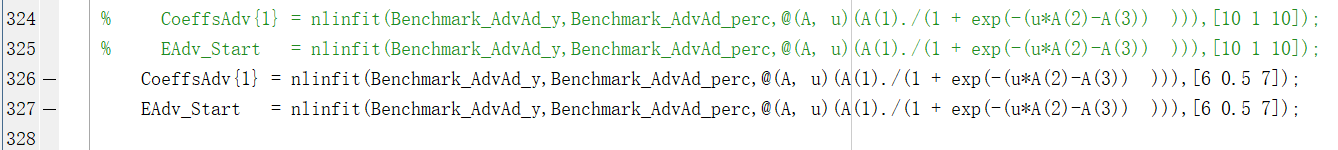


Note: The commented part is the original parameter.

Changed the parameters of progression rate of early adenomas in first run of 1^st^ iteration:


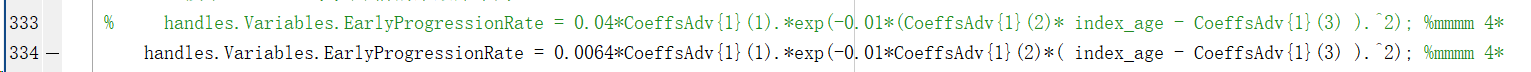


Note: The commented part is the original parameter.


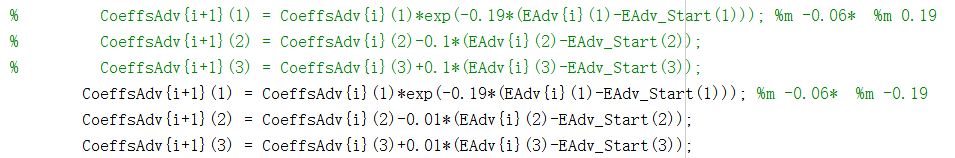
Adjusted the parameters of the coefficients for adenoma progression in numbers ≥2 of the 1^st^ iteration:

- Third, numbers were set 30 for first iteration and 60 for second iteration, following the manual's recommendations.


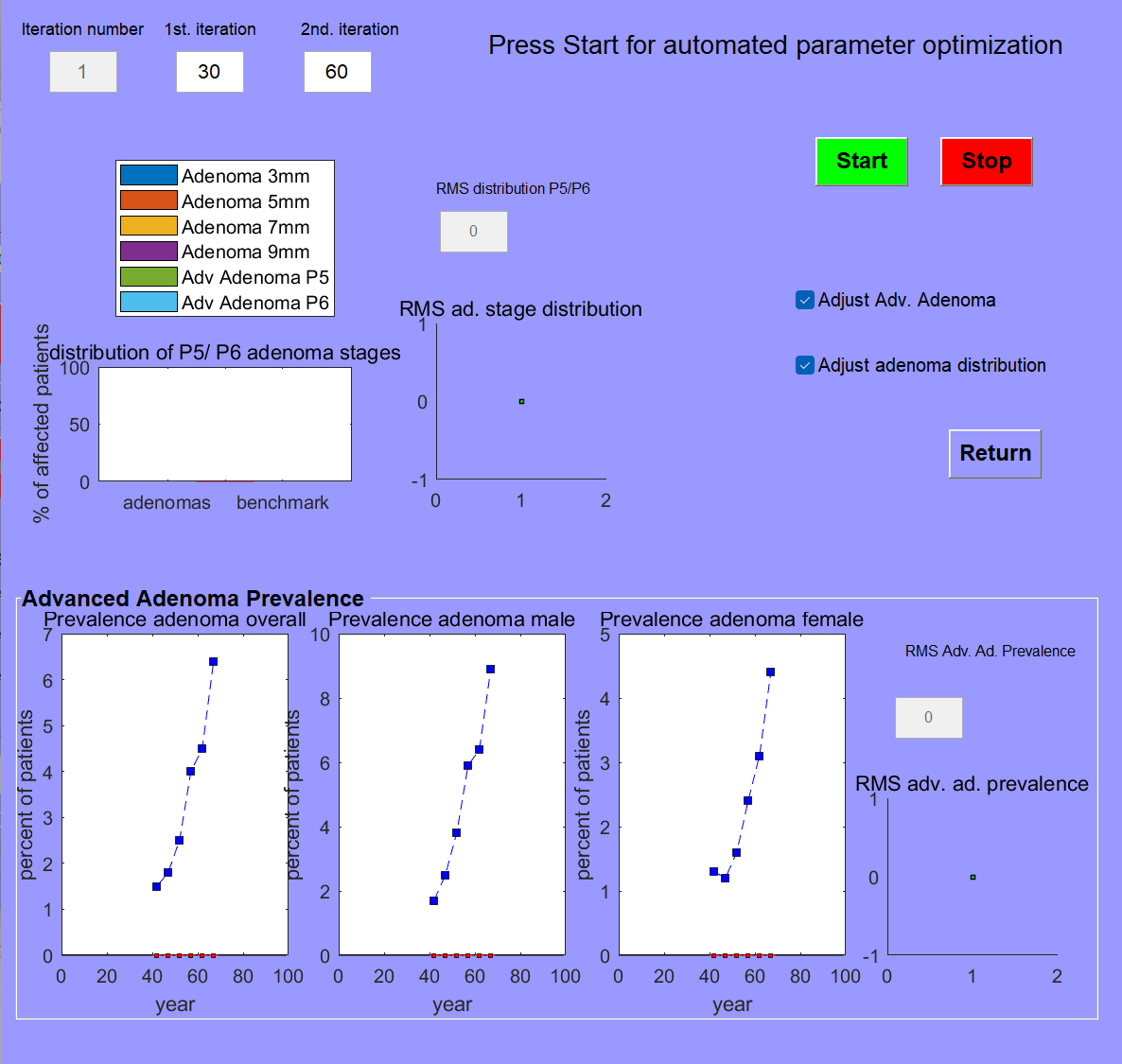


**3. Step 3 (Calibration for cancer incidence)**

We calibrated the model for cancer incidence using the "incidence of CRC in 2019" and the "location of CRC in the rectum" in China as targets. The process involved adjustments to the "Auto_Calibration_Step_3.m" file according to the CMOST manual.

- First, we set a simulated population of 100,000, also disabled flags of CRC screening, adenomas surveillance and cancer surveillance.
- Second, we adjusted the file of “Auto_Calibration_Step_3.m”：

Changed the parameters of the coefficients for adenoma progression in first run of the 1^st^ iteration:


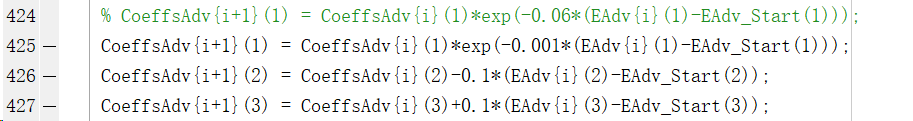
,

Note: The commented part is the original parameter.

and in numbers ≥2 of the 1^st^ iteration:


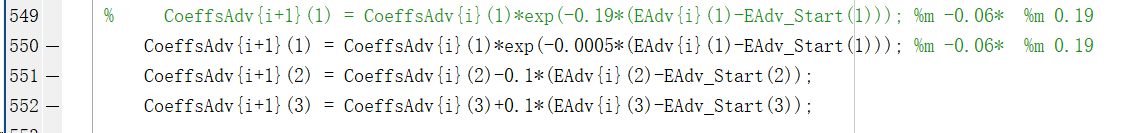
.

Note: The commented part is the original parameter.

Adjusted the parameters of the coefficients for CRC incidence in numbers ≥2 of the 1st iteration:


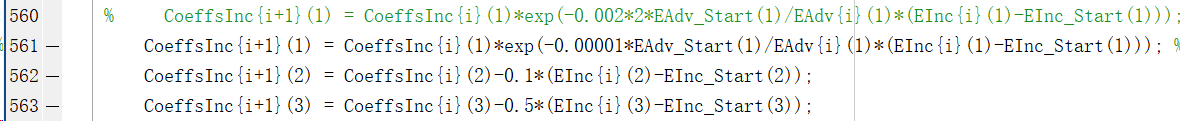
.

Note: The commented part is the original parameter.

Modified the parameter in function of “AdjustRates” for advanced adenomas progress:


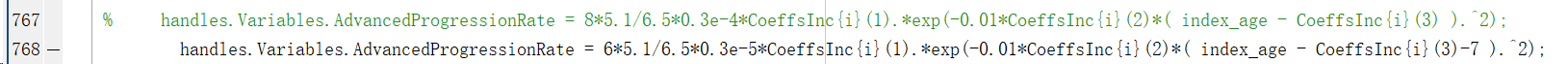


Note: The commented part is the original parameter.

- Third, we adjusted the function of “AdjustRates” in the “Auto_Calib_3_TempFunction” file as it was in the“Auto_Calibration_Step_3.m”:


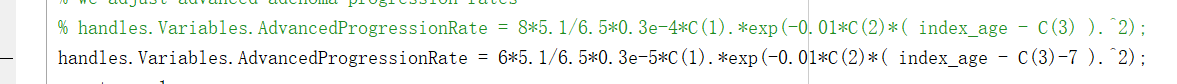


Note: The commented part is the original parameter.

- Lastly, numbers were set 50 for first iteration and 60 for second iteration as suggested by manual.


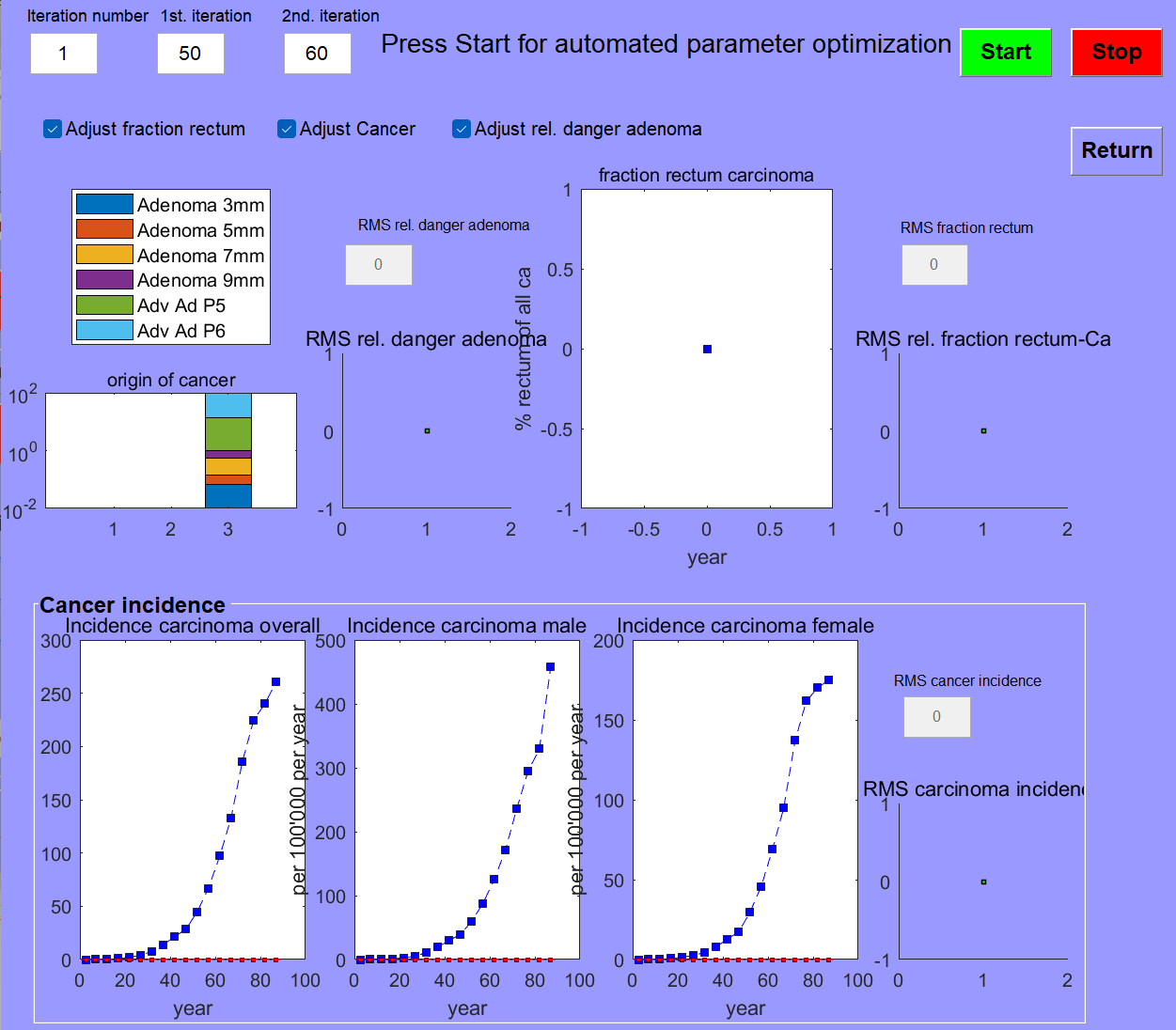

Supplement: Supplementary file 2 — Supplementary Material 2. [file 12889_2024_18201_MOESM2_ESM.docx]
