# Supplementary material for: Efficient organized colorectal cancer screening in Shenzhen: a microsimulation modelling study
Source: BMC Public Health. 2024 Mar 1;24:655. doi: 10.1186/s12889-024-18201-w (PMC10905924; doi:10.1186/s12889-024-18201-w)
Supplement: Supplementary file 3 — Supplementary Material 3. [file 12889_2024_18201_MOESM3_ESM.docx]

**STable 1 Burdens, benefits and harms per 1000 individuals of different screening strategies estimated by the CMOST model (main analysis)**

| Screening strategy | Cols | Non-Col tests | CRC cases | CRC deaths | Reduced incidence | Reduced mortality | LYG | Reduced incidence Rate (%) | Reduced mortality Rate (%) | Patients died of Col | Years lost due to Col |
| --- | --- | --- | --- | --- | --- | --- | --- | --- | --- | --- | --- |
| No screening | 49 | 0 | 46 | 17 | / | / | / | / | / | 0.00 | 0.02 |
| FIT, 40-70, 1 | 2232 | 20461 | 23 | 7 | 23 | 11 | 145 | 49.4 | 61.5 | 0.09 | 2.26 |
| FIT, 40-70, 2 | 1522 | 12444 | 27 | 8 | 20 | 9 | 126 | 42.1 | 54.2 | 0.05 | 1.09 |
| FIT, 40-70, 3 | 1202 | 9064 | 30 | 9 | 17 | 8 | 105 | 36.0 | 45.8 | 0.05 | 1.05 |
| FIT, 40-75, 1 | 2476 | 22938 | 22 | 6 | 24 | 12 | 151 | 51.6 | 66.7 | 0.10 | 2.25 |
| FIT, 40-75, 2 | 1685 | 13954 | 26 | 7 | 21 | 10 | 133 | 44.3 | 59.3 | 0.06 | 1.22 |
| FIT, 40-75, 3 | 1321 | 10116 | 29 | 9 | 18 | 9 | 110 | 38.5 | 50.7 | 0.05 | 1.12 |
| FIT, 40-80, 1 | 2670 | 24963 | 22 | 5 | 24 | 12 | 155 | 52.5 | 71.3 | 0.10 | 2.23 |
| FIT, 40-80, 2 | 1812 | 15196 | 25 | 7 | 21 | 11 | 136 | 45.0 | 62.6 | 0.06 | 1.25 |
| FIT, 40-80, 3 | 1422 | 11029 | 28 | 8 | 18 | 9 | 114 | 39.2 | 54.0 | 0.05 | 1.07 |
| FIT, 45-70, 1 | 1944 | 16974 | 24 | 7 | 22 | 10 | 133 | 47.6 | 59.9 | 0.06 | 1.26 |
| FIT, 45-70, 2 | 1344 | 10342 | 28 | 8 | 19 | 9 | 114 | 40.0 | 51.4 | 0.06 | 1.27 |
| FIT, 45-70, 3 | 1068 | 7542 | 31 | 10 | 16 | 8 | 98 | 34.1 | 44.9 | 0.03 | 0.71 |
| FIT, 45-75, 1 | 2189 | 19448 | 23 | 6 | 23 | 11 | 139 | 49.8 | 64.6 | 0.08 | 1.40 |
| FIT, 45-75, 2 | 1510 | 11858 | 27 | 8 | 20 | 10 | 120 | 42.3 | 56.6 | 0.06 | 1.30 |
| FIT, 45-75, 3 | 1197 | 8660 | 30 | 9 | 17 | 9 | 103 | 36.3 | 49.9 | 0.03 | 0.65 |
| FIT, 45-80, 1 | 2382 | 21470 | 23 | 5 | 23 | 12 | 143 | 50.7 | 69.0 | 0.09 | 1.52 |
| FIT, 45-80, 2 | 1637 | 13093 | 26 | 7 | 20 | 11 | 124 | 43.2 | 60.4 | 0.07 | 1.36 |
| FIT, 45-80, 3 | 1291 | 9523 | 29 | 8 | 17 | 9 | 108 | 37.6 | 53.4 | 0.04 | 0.69 |
| FIT, 50-70, 1 | 1655 | 13544 | 26 | 8 | 21 | 10 | 116 | 44.3 | 56.2 | 0.07 | 1.26 |
| FIT, 50-70, 2 | 1161 | 8299 | 30 | 9 | 17 | 8 | 98 | 36.3 | 47.8 | 0.04 | 0.62 |
| FIT, 50-70, 3 | 920 | 6013 | 32 | 10 | 14 | 7 | 83 | 30.9 | 41.5 | 0.04 | 0.67 |
| FIT, 50-75, 1 | 1899 | 16012 | 25 | 7 | 22 | 11 | 124 | 46.5 | 61.9 | 0.06 | 1.26 |
| FIT, 50-75, 2 | 1322 | 9796 | 28 | 8 | 18 | 9 | 105 | 39.1 | 53.7 | 0.05 | 0.62 |
| FIT, 50-75, 3 | 1055 | 7165 | 31 | 9 | 16 | 8 | 90 | 34.1 | 47.4 | 0.04 | 0.80 |
| FIT, 50-80, 1 | 2091 | 18034 | 25 | 6 | 22 | 11 | 126 | 47.1 | 65.6 | 0.07 | 1.19 |
| FIT, 50-80, 2 | 1452 | 11037 | 28 | 8 | 18 | 10 | 108 | 39.6 | 56.9 | 0.05 | 0.76 |
| FIT, 50-80, 3 | 1155 | 8078 | 30 | 9 | 16 | 9 | 93 | 34.5 | 50.5 | 0.04 | 0.84 |
| NCPCS, 40-70, 1 | 4107 | 14715 | 21 | 7 | 25 | 11 | 141 | 54.4 | 62.0 | 0.13 | 3.23 |
| NCPCS, 40-70, 2 | 3107 | 10553 | 23 | 7 | 24 | 10 | 130 | 50.9 | 58.1 | 0.12 | 2.66 |
| NCPCS, 40-70, 3 | 2542 | 8294 | 24 | 8 | 22 | 9 | 119 | 47.5 | 53.7 | 0.10 | 2.35 |
| NCPCS, 40-75, 1 | 4565 | 16440 | 20 | 6 | 26 | 12 | 145 | 56.8 | 65.9 | 0.15 | 3.32 |
| NCPCS, 40-75, 2 | 3450 | 11801 | 22 | 7 | 25 | 11 | 132 | 53.2 | 61.2 | 0.12 | 2.85 |
| NCPCS, 40-75, 3 | 2822 | 9284 | 23 | 8 | 23 | 10 | 122 | 49.5 | 56.5 | 0.11 | 2.41 |
| NCPCS, 40-80, 1 | 4930 | 17855 | 20 | 6 | 27 | 12 | 148 | 57.3 | 67.8 | 0.16 | 3.41 |
| NCPCS, 40-80, 2 | 3722 | 12824 | 21 | 6 | 25 | 11 | 135 | 54.1 | 63.8 | 0.12 | 2.79 |
| NCPCS, 40-80, 3 | 3044 | 10090 | 23 | 7 | 23 | 10 | 125 | 50.5 | 59.0 | 0.12 | 2.63 |
| NCPCS, 45-70, 1 | 3523 | 12216 | 22 | 7 | 24 | 10 | 126 | 52.3 | 58.9 | 0.13 | 3.06 |
| NCPCS, 45-70, 2 | 2684 | 8770 | 24 | 8 | 23 | 10 | 115 | 48.7 | 55.2 | 0.09 | 2.03 |
| NCPCS, 45-70, 3 | 2213 | 6912 | 26 | 9 | 21 | 9 | 103 | 44.5 | 50.3 | 0.06 | 1.36 |
| NCPCS, 45-75, 1 | 3980 | 13938 | 21 | 7 | 25 | 11 | 131 | 54.2 | 62.6 | 0.14 | 3.09 |
| NCPCS, 45-75, 2 | 3026 | 10017 | 23 | 7 | 24 | 10 | 119 | 50.9 | 58.6 | 0.10 | 2.16 |
| NCPCS, 45-75, 3 | 2492 | 7893 | 24 | 8 | 22 | 10 | 109 | 47.2 | 54.7 | 0.07 | 1.51 |
| NCPCS, 45-80, 1 | 4343 | 15352 | 21 | 6 | 26 | 11 | 133 | 55.1 | 65.3 | 0.15 | 3.18 |
| NCPCS, 45-80, 2 | 3297 | 11037 | 22 | 7 | 24 | 11 | 121 | 52.1 | 61.2 | 0.11 | 2.20 |
| NCPCS, 45-80, 3 | 2714 | 8703 | 24 | 8 | 22 | 10 | 110 | 47.9 | 55.9 | 0.08 | 1.60 |
| NCPCS, 50-70, 1 | 2939 | 9758 | 23 | 8 | 23 | 10 | 115 | 49.5 | 56.6 | 0.09 | 1.98 |
| NCPCS, 50-70, 2 | 2260 | 7031 | 25 | 8 | 21 | 9 | 103 | 45.5 | 51.9 | 0.07 | 1.40 |
| NCPCS, 50-70, 3 | 1880 | 5585 | 27 | 9 | 19 | 8 | 93 | 41.5 | 48.3 | 0.07 | 1.32 |
| NCPCS, 50-75, 1 | 3395 | 11476 | 23 | 7 | 24 | 11 | 118 | 51.2 | 60.3 | 0.11 | 2.18 |
| NCPCS, 50-75, 2 | 2601 | 8271 | 24 | 8 | 22 | 10 | 105 | 47.4 | 55.4 | 0.09 | 1.56 |
| NCPCS, 50-75, 3 | 2152 | 6537 | 26 | 8 | 20 | 9 | 97 | 43.9 | 51.4 | 0.07 | 1.36 |
| NCPCS, 50-80, 1 | 3757 | 12884 | 22 | 7 | 24 | 11 | 120 | 52.4 | 62.7 | 0.12 | 2.25 |
| NCPCS, 50-80, 2 | 2872 | 9289 | 24 | 7 | 23 | 10 | 108 | 48.6 | 58.2 | 0.10 | 1.72 |
| NCPCS, 50-80, 3 | 2370 | 7338 | 25 | 8 | 21 | 9 | 98 | 45.1 | 53.6 | 0.08 | 1.42 |
| RS model, 40-70, 1 | 4917 | 11006 | 20 | 6 | 26 | 11 | 148 | 56.0 | 63.6 | 0.16 | 4.20 |
| RS model, 40-70, 2 | 4091 | 8864 | 21 | 7 | 25 | 11 | 145 | 54.6 | 62.3 | 0.12 | 3.21 |
| RS model, 40-70, 3 | 3539 | 7475 | 22 | 7 | 24 | 10 | 138 | 52.6 | 60.0 | 0.11 | 2.60 |
| RS model, 40-75, 1 | 5464 | 12280 | 19 | 6 | 27 | 12 | 152 | 58.0 | 67.7 | 0.19 | 4.48 |
| RS model, 40-75, 2 | 4543 | 9899 | 20 | 6 | 26 | 12 | 151 | 57.1 | 67.4 | 0.14 | 3.50 |
| RS model, 40-75, 3 | 3928 | 8346 | 21 | 6 | 26 | 11 | 145 | 55.0 | 64.3 | 0.12 | 2.83 |
| RS model, 40-80, 1 | 5901 | 13325 | 19 | 5 | 27 | 12 | 154 | 59.0 | 70.6 | 0.20 | 4.73 |
| RS model, 40-80, 2 | 4903 | 10748 | 20 | 5 | 27 | 12 | 152 | 57.6 | 69.3 | 0.14 | 3.39 |
| RS model, 40-80, 3 | 4237 | 9060 | 20 | 6 | 26 | 12 | 146 | 56.0 | 67.2 | 0.13 | 2.87 |
| RS model, 45-70, 1 | 4204 | 9132 | 21 | 7 | 25 | 11 | 137 | 53.9 | 62.4 | 0.12 | 2.69 |
| RS model, 45-70, 2 | 3511 | 7364 | 22 | 7 | 25 | 11 | 132 | 52.9 | 60.8 | 0.12 | 2.92 |
| RS model, 45-70, 3 | 3054 | 6220 | 23 | 7 | 24 | 10 | 128 | 51.4 | 58.6 | 0.12 | 2.53 |
| RS model, 45-75, 1 | 4749 | 10406 | 20 | 6 | 26 | 12 | 142 | 55.8 | 66.1 | 0.14 | 3.02 |
| RS model, 45-75, 2 | 3963 | 8397 | 21 | 6 | 25 | 11 | 135 | 54.7 | 64.3 | 0.13 | 2.94 |
| RS model, 45-75, 3 | 3441 | 7088 | 21 | 6 | 25 | 11 | 131 | 53.6 | 62.9 | 0.13 | 2.64 |
| RS model, 45-80, 1 | 5186 | 11449 | 20 | 5 | 26 | 12 | 144 | 56.5 | 68.8 | 0.15 | 3.10 |
| RS model, 45-80, 2 | 4324 | 9244 | 21 | 6 | 26 | 12 | 137 | 55.3 | 66.5 | 0.15 | 3.02 |
| RS model, 45-80, 3 | 3747 | 7799 | 21 | 6 | 25 | 11 | 134 | 54.5 | 65.6 | 0.14 | 2.72 |
| RS model, 50-70, 1 | 3494 | 7308 | 23 | 7 | 23 | 10 | 121 | 50.3 | 58.4 | 0.11 | 2.11 |
| RS model, 50-70, 2 | 2933 | 5895 | 24 | 7 | 23 | 10 | 117 | 49.3 | 57.7 | 0.09 | 2.03 |
| RS model, 50-70, 3 | 2564 | 4992 | 24 | 8 | 22 | 10 | 114 | 47.7 | 55.9 | 0.08 | 1.47 |
| RS model, 50-75, 1 | 4037 | 8576 | 22 | 7 | 24 | 11 | 125 | 52.2 | 62.3 | 0.12 | 2.20 |
| RS model, 50-75, 2 | 3384 | 6929 | 22 | 7 | 24 | 11 | 121 | 51.5 | 61.7 | 0.10 | 2.31 |
| RS model, 50-75, 3 | 2955 | 5865 | 23 | 7 | 23 | 10 | 118 | 49.9 | 59.8 | 0.09 | 1.74 |
| RS model, 50-80, 1 | 4470 | 9618 | 22 | 6 | 25 | 11 | 128 | 53.0 | 64.9 | 0.13 | 2.44 |
| RS model, 50-80, 2 | 3744 | 7773 | 22 | 6 | 24 | 11 | 123 | 52.5 | 64.1 | 0.12 | 2.44 |
| RS model, 50-80, 3 | 3261 | 6576 | 23 | 7 | 24 | 11 | 119 | 50.9 | 62.0 | 0.10 | 1.76 |

**Abbreviations:** COL, colonoscopy; CRC, colorectal cancer; LYG, life-years gain; FIT, fecal immunochemical test; NCPCS, National Colorectal Polyp Care score; RS, Risk-stratification.

**STable 2 Burdens, benefits and harms per 1000 individuals of different screening strategies estimated by the CMOST model (low participation rates)**

| Screening strategy | Cols | Non-Col tests | CRC cases | CRC deaths | Reduced incidence | Reduced mortality | LYG | Reduced incidence Rate (%) | Reduced mortality Rate (%) | Patients died of Col | Years lost due to Col |
| --- | --- | --- | --- | --- | --- | --- | --- | --- | --- | --- | --- |
| FIT, 40-70, 1 | 1198 | 10697 | 29 | 9 | 17 | 8 | 107 | 36.4 | 47.2 | 0.04 | 0.95 |
| FIT, 40-70, 2 | 969 | 7996 | 32 | 10 | 14 | 7 | 89 | 30.8 | 40.1 | 0.04 | 0.76 |
| FIT, 40-70, 3 | 829 | 6432 | 34 | 11 | 13 | 6 | 77 | 27.3 | 34.7 | 0.03 | 0.58 |
| FIT, 40-75, 1 | 1327 | 12029 | 29 | 9 | 18 | 9 | 111 | 38.3 | 51.4 | 0.04 | 0.83 |
| FIT, 40-75, 2 | 1071 | 8993 | 31 | 10 | 15 | 8 | 96 | 32.6 | 44.3 | 0.04 | 0.79 |
| FIT, 40-75, 3 | 913 | 7229 | 33 | 11 | 14 | 7 | 82 | 29.3 | 39.1 | 0.04 | 0.62 |
| FIT, 40-80, 1 | 1428 | 13121 | 28 | 8 | 18 | 9 | 114 | 39.0 | 53.9 | 0.04 | 0.95 |
| FIT, 40-80, 2 | 1150 | 9810 | 31 | 9 | 16 | 8 | 98 | 33.8 | 46.9 | 0.04 | 0.77 |
| FIT, 40-80, 3 | 977 | 7884 | 32 | 10 | 14 | 7 | 83 | 30.2 | 41.6 | 0.04 | 0.77 |
| FIT, 45-70, 1 | 1065 | 8877 | 31 | 10 | 15 | 7 | 91 | 33.0 | 42.1 | 0.04 | 0.72 |
| FIT, 45-70, 2 | 862 | 6650 | 33 | 11 | 13 | 7 | 78 | 29.1 | 37.4 | 0.04 | 0.72 |
| FIT, 45-70, 3 | 744 | 5362 | 35 | 12 | 11 | 6 | 66 | 24.7 | 32.3 | 0.04 | 0.61 |
| FIT, 45-75, 1 | 1194 | 10209 | 30 | 9 | 17 | 8 | 98 | 35.7 | 48.1 | 0.05 | 0.83 |
| FIT, 45-75, 2 | 964 | 7646 | 32 | 10 | 14 | 7 | 83 | 31.1 | 41.4 | 0.04 | 0.77 |
| FIT, 45-75, 3 | 828 | 6160 | 34 | 11 | 13 | 6 | 73 | 27.5 | 37.1 | 0.04 | 0.73 |
| FIT, 45-80, 1 | 1294 | 11301 | 29 | 9 | 17 | 9 | 102 | 36.6 | 51.2 | 0.05 | 0.89 |
| FIT, 45-80, 2 | 1044 | 8464 | 32 | 10 | 15 | 8 | 86 | 32.0 | 44.8 | 0.04 | 0.71 |
| FIT, 45-80, 3 | 893 | 6815 | 33 | 11 | 13 | 7 | 74 | 28.3 | 39.1 | 0.04 | 0.75 |
| FIT, 50-70, 1 | 917 | 7086 | 32 | 10 | 14 | 7 | 80 | 30.5 | 40.2 | 0.04 | 0.57 |
| FIT, 50-70, 2 | 754 | 5325 | 34 | 12 | 12 | 6 | 67 | 26.0 | 34.2 | 0.03 | 0.48 |
| FIT, 50-70, 3 | 653 | 4306 | 36 | 12 | 10 | 5 | 59 | 22.1 | 29.2 | 0.03 | 0.45 |
| FIT, 50-75, 1 | 1046 | 8417 | 31 | 10 | 16 | 8 | 86 | 33.7 | 45.1 | 0.05 | 0.71 |
| FIT, 50-75, 2 | 859 | 6323 | 33 | 11 | 13 | 7 | 71 | 27.8 | 39.2 | 0.04 | 0.44 |
| FIT, 50-75, 3 | 738 | 5104 | 35 | 12 | 11 | 6 | 64 | 24.7 | 34.2 | 0.04 | 0.62 |
| FIT, 50-80, 1 | 1148 | 9508 | 30 | 9 | 16 | 9 | 90 | 34.6 | 49.1 | 0.05 | 0.84 |
| FIT, 50-80, 2 | 937 | 7140 | 33 | 10 | 13 | 7 | 73 | 28.8 | 41.6 | 0.04 | 0.46 |
| FIT, 50-80, 3 | 804 | 5761 | 34 | 11 | 12 | 7 | 67 | 25.8 | 37.7 | 0.04 | 0.61 |
| NCPCS, 40-70, 1 | 1034 | 3995 | 35 | 12 | 12 | 5 | 58 | 25.0 | 28.7 | 0.03 | 0.54 |
| NCPCS, 40-70, 2 | 953 | 3589 | 35 | 13 | 11 | 5 | 51 | 23.5 | 25.9 | 0.04 | 0.71 |
| NCPCS, 40-70, 3 | 886 | 3268 | 36 | 13 | 10 | 4 | 49 | 22.0 | 24.8 | 0.03 | 0.72 |
| NCPCS, 40-75, 1 | 1144 | 4498 | 34 | 12 | 13 | 5 | 59 | 27.2 | 30.6 | 0.04 | 0.57 |
| NCPCS, 40-75, 2 | 1055 | 4040 | 34 | 12 | 12 | 5 | 55 | 25.7 | 29.7 | 0.04 | 0.77 |
| NCPCS, 40-75, 3 | 978 | 3680 | 35 | 13 | 11 | 5 | 53 | 23.9 | 27.2 | 0.03 | 0.69 |
| NCPCS, 40-80, 1 | 1230 | 4912 | 33 | 12 | 13 | 6 | 60 | 28.2 | 32.3 | 0.04 | 0.69 |
| NCPCS, 40-80, 2 | 1134 | 4413 | 34 | 12 | 12 | 5 | 54 | 26.2 | 29.7 | 0.05 | 0.82 |
| NCPCS, 40-80, 3 | 1050 | 4018 | 35 | 12 | 12 | 5 | 54 | 24.9 | 28.7 | 0.04 | 0.73 |
| NCPCS, 45-70, 1 | 912 | 3318 | 35 | 13 | 11 | 5 | 51 | 23.6 | 26.5 | 0.04 | 0.57 |
| NCPCS, 45-70, 2 | 841 | 2983 | 36 | 13 | 10 | 4 | 48 | 21.6 | 25.6 | 0.03 | 0.52 |
| NCPCS, 45-70, 3 | 784 | 2719 | 37 | 13 | 9 | 4 | 41 | 20.2 | 23.1 | 0.02 | 0.43 |
| NCPCS, 45-75, 1 | 1024 | 3821 | 35 | 12 | 12 | 5 | 54 | 25.4 | 30.0 | 0.04 | 0.68 |
| NCPCS, 45-75, 2 | 942 | 3436 | 35 | 13 | 11 | 5 | 51 | 23.6 | 28.0 | 0.03 | 0.52 |
| NCPCS, 45-75, 3 | 877 | 3131 | 36 | 13 | 10 | 4 | 44 | 21.8 | 25.2 | 0.03 | 0.51 |
| NCPCS, 45-80, 1 | 1111 | 4235 | 34 | 12 | 12 | 6 | 56 | 26.5 | 31.7 | 0.04 | 0.71 |
| NCPCS, 45-80, 2 | 1021 | 3808 | 35 | 12 | 11 | 5 | 51 | 24.5 | 29.0 | 0.03 | 0.54 |
| NCPCS, 45-80, 3 | 948 | 3469 | 36 | 13 | 10 | 5 | 45 | 22.6 | 26.3 | 0.03 | 0.49 |
| NCPCS, 50-70, 1 | 780 | 2651 | 37 | 13 | 9 | 4 | 40 | 20.5 | 22.9 | 0.02 | 0.33 |
| NCPCS, 50-70, 2 | 726 | 2384 | 37 | 14 | 9 | 4 | 39 | 19.2 | 22.5 | 0.02 | 0.39 |
| NCPCS, 50-70, 3 | 680 | 2178 | 38 | 14 | 8 | 4 | 36 | 18.0 | 20.3 | 0.01 | 0.27 |
| NCPCS, 50-75, 1 | 893 | 3154 | 36 | 13 | 10 | 5 | 44 | 22.6 | 26.6 | 0.03 | 0.38 |
| NCPCS, 50-75, 2 | 828 | 2837 | 37 | 13 | 10 | 4 | 41 | 20.8 | 24.9 | 0.03 | 0.48 |
| NCPCS, 50-75, 3 | 773 | 2589 | 37 | 14 | 9 | 4 | 38 | 19.2 | 21.9 | 0.03 | 0.37 |
| NCPCS, 50-80, 1 | 980 | 3568 | 35 | 13 | 11 | 5 | 45 | 23.4 | 27.6 | 0.03 | 0.38 |
| NCPCS, 50-80, 2 | 907 | 3209 | 36 | 13 | 10 | 5 | 43 | 22.3 | 26.6 | 0.03 | 0.48 |
| NCPCS, 50-80, 3 | 845 | 2927 | 37 | 13 | 9 | 4 | 39 | 20.2 | 23.6 | 0.03 | 0.39 |
| RS model, 40-70, 1 | 1114 | 3596 | 34 | 12 | 13 | 5 | 64 | 27.0 | 30.7 | 0.05 | 1.26 |
| RS model, 40-70, 2 | 1033 | 3275 | 34 | 12 | 12 | 5 | 59 | 25.8 | 29.8 | 0.05 | 1.13 |
| RS model, 40-70, 3 | 973 | 3018 | 35 | 13 | 11 | 5 | 59 | 23.7 | 28.0 | 0.04 | 1.03 |
| RS model, 40-75, 1 | 1235 | 4048 | 33 | 12 | 13 | 6 | 66 | 28.4 | 33.9 | 0.06 | 1.30 |
| RS model, 40-75, 2 | 1146 | 3687 | 34 | 12 | 13 | 6 | 62 | 27.6 | 32.3 | 0.06 | 1.30 |
| RS model, 40-75, 3 | 1074 | 3396 | 34 | 12 | 12 | 5 | 61 | 26.1 | 31.2 | 0.05 | 1.04 |
| RS model, 40-80, 1 | 1329 | 4418 | 33 | 11 | 14 | 6 | 66 | 29.3 | 35.3 | 0.05 | 1.24 |
| RS model, 40-80, 2 | 1232 | 4025 | 33 | 12 | 13 | 6 | 64 | 28.2 | 33.8 | 0.06 | 1.29 |
| RS model, 40-80, 3 | 1154 | 3706 | 34 | 12 | 12 | 6 | 63 | 26.7 | 32.1 | 0.05 | 1.02 |
| RS model, 45-70, 1 | 977 | 2987 | 35 | 13 | 12 | 5 | 54 | 24.9 | 28.2 | 0.03 | 0.64 |
| RS model, 45-70, 2 | 913 | 2721 | 36 | 13 | 11 | 5 | 54 | 23.3 | 27.4 | 0.04 | 0.70 |
| RS model, 45-70, 3 | 859 | 2508 | 36 | 13 | 10 | 5 | 51 | 22.0 | 26.2 | 0.03 | 0.49 |
| RS model, 45-75, 1 | 1098 | 3438 | 34 | 12 | 12 | 5 | 57 | 26.6 | 31.2 | 0.04 | 0.71 |
| RS model, 45-75, 2 | 1024 | 3132 | 35 | 12 | 12 | 5 | 55 | 25.2 | 30.3 | 0.04 | 0.64 |
| RS model, 45-75, 3 | 962 | 2886 | 35 | 13 | 11 | 5 | 52 | 23.6 | 28.2 | 0.04 | 0.63 |
| RS model, 45-80, 1 | 1193 | 3809 | 34 | 12 | 13 | 6 | 59 | 27.7 | 32.9 | 0.04 | 0.73 |
| RS model, 45-80, 2 | 1111 | 3470 | 34 | 12 | 12 | 6 | 56 | 26.0 | 31.6 | 0.05 | 0.76 |
| RS model, 45-80, 3 | 1041 | 3197 | 35 | 12 | 11 | 5 | 54 | 24.8 | 30.1 | 0.04 | 0.61 |
| RS model, 50-70, 1 | 839 | 2381 | 36 | 13 | 10 | 5 | 45 | 21.7 | 25.8 | 0.03 | 0.41 |
| RS model, 50-70, 2 | 785 | 2174 | 37 | 13 | 9 | 4 | 45 | 20.4 | 24.6 | 0.03 | 0.68 |
| RS model, 50-70, 3 | 741 | 2007 | 37 | 13 | 9 | 4 | 42 | 19.5 | 23.4 | 0.03 | 0.53 |
| RS model, 50-75, 1 | 961 | 2834 | 35 | 12 | 11 | 5 | 48 | 24.0 | 28.6 | 0.03 | 0.46 |
| RS model, 50-75, 2 | 897 | 2586 | 36 | 13 | 10 | 5 | 47 | 22.0 | 26.6 | 0.04 | 0.70 |
| RS model, 50-75, 3 | 844 | 2386 | 36 | 13 | 10 | 5 | 44 | 21.4 | 26.3 | 0.04 | 0.51 |
| RS model, 50-80, 1 | 1057 | 3206 | 35 | 12 | 12 | 5 | 50 | 25.0 | 30.9 | 0.04 | 0.52 |
| RS model, 50-80, 2 | 985 | 2924 | 36 | 12 | 11 | 5 | 48 | 23.3 | 29.5 | 0.04 | 0.76 |
| RS model, 50-80, 3 | 925 | 2698 | 36 | 13 | 10 | 5 | 45 | 22.3 | 26.9 | 0.04 | 0.54 |

**Abbreviations:** COL, colonoscopy; CRC, colorectal cancer; LYG, life-years gain; FIT, fecal immunochemical test; NCPCS, National Colorectal Polyp Care score; RS, Risk-stratification.

**STable 3 Burdens, benefits and harms per 1000 individuals of different screening strategies estimated by the CMOST model (no surveillance colonoscopy)**

| Screening strategy | Cols | Non-Col tests | CRC cases | CRC deaths | Reduced incidence | Reduced mortality | LYG | Reduced incidence Rate (%) | Reduced mortality Rate (%) | Patients died of Col | Years lost due to Col |
| --- | --- | --- | --- | --- | --- | --- | --- | --- | --- | --- | --- |
| FIT, 40-70, 1 | 1703 | 21441 | 27 | 8 | 19 | 9 | 136 | 41.0 | 53.2 | 0.06 | 1.45 |
| FIT, 40-70, 2 | 1053 | 12896 | 31 | 9 | 15 | 8 | 117 | 33.4 | 45.8 | 0.04 | 0.97 |
| FIT, 40-70, 3 | 782 | 9342 | 34 | 11 | 13 | 6 | 93 | 27.0 | 36.8 | 0.03 | 0.73 |
| FIT, 40-75, 1 | 1926 | 24233 | 25 | 7 | 21 | 11 | 147 | 45.4 | 62.1 | 0.07 | 1.48 |
| FIT, 40-75, 2 | 1188 | 14565 | 29 | 8 | 17 | 9 | 125 | 37.0 | 52.6 | 0.04 | 1.02 |
| FIT, 40-75, 3 | 877 | 10496 | 32 | 10 | 14 | 8 | 103 | 30.5 | 44.3 | 0.03 | 0.74 |
| FIT, 40-80, 1 | 2114 | 26563 | 24 | 6 | 22 | 12 | 152 | 47.3 | 67.9 | 0.08 | 1.57 |
| FIT, 40-80, 2 | 1304 | 15961 | 28 | 7 | 18 | 10 | 129 | 38.5 | 57.9 | 0.05 | 1.11 |
| FIT, 40-80, 3 | 963 | 11502 | 31 | 9 | 15 | 8 | 108 | 32.1 | 48.6 | 0.03 | 0.78 |
| FIT, 45-70, 1 | 1431 | 17856 | 28 | 8 | 18 | 9 | 125 | 39.4 | 51.5 | 0.06 | 1.44 |
| FIT, 45-70, 2 | 892 | 10754 | 32 | 10 | 14 | 7 | 102 | 30.8 | 41.8 | 0.03 | 0.89 |
| FIT, 45-70, 3 | 663 | 7782 | 34 | 11 | 12 | 6 | 85 | 25.8 | 35.4 | 0.03 | 0.68 |
| FIT, 45-75, 1 | 1653 | 20645 | 26 | 7 | 20 | 10 | 135 | 43.6 | 59.8 | 0.06 | 1.50 |
| FIT, 45-75, 2 | 1028 | 12427 | 30 | 9 | 16 | 9 | 112 | 34.9 | 50.3 | 0.04 | 0.94 |
| FIT, 45-75, 3 | 765 | 9010 | 33 | 10 | 13 | 7 | 92 | 28.8 | 41.6 | 0.03 | 0.68 |
| FIT, 45-80, 1 | 1840 | 22978 | 25 | 6 | 21 | 11 | 140 | 45.5 | 65.3 | 0.07 | 1.58 |
| FIT, 45-80, 2 | 1143 | 13823 | 29 | 8 | 17 | 10 | 116 | 36.6 | 54.9 | 0.04 | 1.03 |
| FIT, 45-80, 3 | 847 | 9974 | 32 | 9 | 14 | 8 | 95 | 29.9 | 45.7 | 0.03 | 0.75 |
| FIT, 50-70, 1 | 1165 | 14285 | 30 | 9 | 17 | 8 | 108 | 35.9 | 48.5 | 0.03 | 0.64 |
| FIT, 50-70, 2 | 732 | 8635 | 33 | 11 | 13 | 7 | 88 | 28.4 | 39.7 | 0.04 | 0.75 |
| FIT, 50-70, 3 | 545 | 6228 | 36 | 12 | 11 | 6 | 76 | 23.3 | 32.6 | 0.02 | 0.48 |
| FIT, 50-75, 1 | 1387 | 17074 | 28 | 8 | 18 | 10 | 117 | 39.6 | 56.4 | 0.04 | 0.70 |
| FIT, 50-75, 2 | 867 | 10298 | 31 | 9 | 15 | 8 | 97 | 32.6 | 46.9 | 0.04 | 0.82 |
| FIT, 50-75, 3 | 647 | 7461 | 34 | 11 | 12 | 7 | 82 | 26.7 | 39.8 | 0.02 | 0.52 |
| FIT, 50-80, 1 | 1575 | 19404 | 27 | 7 | 19 | 11 | 123 | 41.7 | 62.3 | 0.05 | 0.85 |
| FIT, 50-80, 2 | 983 | 11695 | 31 | 8 | 16 | 9 | 102 | 34.1 | 52.6 | 0.04 | 0.89 |
| FIT, 50-80, 3 | 734 | 8486 | 33 | 10 | 13 | 8 | 86 | 28.1 | 43.9 | 0.03 | 0.54 |
| NCPCS, 40-70, 1 | 3615 | 15712 | 24 | 8 | 22 | 9 | 130 | 47.7 | 53.7 | 0.11 | 2.98 |
| NCPCS, 40-70, 2 | 2594 | 11166 | 27 | 9 | 20 | 8 | 116 | 42.2 | 48.0 | 0.08 | 2.33 |
| NCPCS, 40-70, 3 | 2040 | 8718 | 29 | 10 | 18 | 8 | 104 | 38.4 | 43.9 | 0.07 | 1.71 |
| NCPCS, 40-75, 1 | 4076 | 17715 | 22 | 7 | 24 | 10 | 137 | 51.6 | 59.6 | 0.13 | 3.37 |
| NCPCS, 40-75, 2 | 2927 | 12595 | 25 | 8 | 21 | 9 | 122 | 46.2 | 54.1 | 0.10 | 2.46 |
| NCPCS, 40-75, 3 | 2303 | 9843 | 27 | 9 | 20 | 9 | 110 | 42.2 | 48.7 | 0.08 | 1.74 |
| NCPCS, 40-80, 1 | 4463 | 19391 | 21 | 6 | 25 | 11 | 142 | 54.4 | 64.8 | 0.15 | 3.38 |
| NCPCS, 40-80, 2 | 3206 | 13791 | 24 | 7 | 23 | 10 | 126 | 48.8 | 58.3 | 0.11 | 2.65 |
| NCPCS, 40-80, 3 | 2521 | 10775 | 26 | 8 | 21 | 9 | 114 | 44.6 | 52.9 | 0.09 | 1.85 |
| NCPCS, 45-70, 1 | 3038 | 13134 | 25 | 8 | 21 | 9 | 118 | 45.8 | 51.4 | 0.10 | 2.15 |
| NCPCS, 45-70, 2 | 2184 | 9337 | 27 | 10 | 19 | 8 | 105 | 40.8 | 45.1 | 0.06 | 1.37 |
| NCPCS, 45-70, 3 | 1725 | 7307 | 30 | 10 | 17 | 7 | 91 | 36.0 | 40.6 | 0.06 | 1.39 |
| NCPCS, 45-75, 1 | 3499 | 15140 | 23 | 7 | 23 | 10 | 126 | 49.7 | 57.1 | 0.11 | 2.43 |
| NCPCS, 45-75, 2 | 2515 | 10765 | 25 | 8 | 21 | 9 | 112 | 45.3 | 52.1 | 0.08 | 1.68 |
| NCPCS, 45-75, 3 | 1984 | 8412 | 28 | 9 | 19 | 8 | 97 | 40.0 | 45.7 | 0.07 | 1.38 |
| NCPCS, 45-80, 1 | 3887 | 16817 | 22 | 7 | 24 | 11 | 129 | 52.4 | 62.7 | 0.12 | 2.55 |
| NCPCS, 45-80, 2 | 2793 | 11958 | 24 | 8 | 22 | 10 | 115 | 47.4 | 55.6 | 0.10 | 1.78 |
| NCPCS, 45-80, 3 | 2204 | 9352 | 27 | 9 | 19 | 9 | 100 | 42.0 | 49.4 | 0.08 | 1.46 |
| NCPCS, 50-70, 1 | 2461 | 10570 | 27 | 9 | 20 | 8 | 103 | 42.8 | 48.3 | 0.07 | 1.80 |
| NCPCS, 50-70, 2 | 1775 | 7520 | 29 | 10 | 17 | 7 | 89 | 37.0 | 42.2 | 0.06 | 1.41 |
| NCPCS, 50-70, 3 | 1415 | 5938 | 31 | 11 | 15 | 7 | 78 | 33.1 | 37.7 | 0.05 | 1.16 |
| NCPCS, 50-75, 1 | 2923 | 12568 | 25 | 8 | 22 | 9 | 110 | 46.7 | 54.1 | 0.09 | 1.98 |
| NCPCS, 50-75, 2 | 2105 | 8945 | 27 | 9 | 19 | 8 | 95 | 41.4 | 48.0 | 0.07 | 1.46 |
| NCPCS, 50-75, 3 | 1666 | 7009 | 29 | 10 | 17 | 7 | 83 | 36.8 | 42.6 | 0.07 | 1.36 |
| NCPCS, 50-80, 1 | 3310 | 14241 | 24 | 7 | 23 | 10 | 115 | 48.8 | 58.5 | 0.11 | 2.10 |
| NCPCS, 50-80, 2 | 2384 | 10139 | 26 | 8 | 20 | 9 | 99 | 43.5 | 52.4 | 0.08 | 1.65 |
| NCPCS, 50-80, 3 | 1883 | 7934 | 28 | 9 | 18 | 8 | 87 | 38.7 | 46.4 | 0.08 | 1.50 |
| RS model, 40-70, 1 | 4469 | 11824 | 23 | 8 | 23 | 10 | 140 | 49.8 | 56.9 | 0.14 | 3.72 |
| RS model, 40-70, 2 | 3598 | 9472 | 24 | 8 | 22 | 9 | 134 | 47.6 | 54.2 | 0.13 | 3.50 |
| RS model, 40-70, 3 | 3036 | 7956 | 25 | 8 | 21 | 9 | 128 | 46.0 | 53.0 | 0.10 | 2.69 |
| RS model, 40-75, 1 | 5035 | 13321 | 21 | 6 | 25 | 11 | 148 | 54.2 | 63.8 | 0.16 | 4.15 |
| RS model, 40-75, 2 | 4056 | 10675 | 22 | 7 | 24 | 11 | 143 | 52.1 | 62.0 | 0.16 | 3.85 |
| RS model, 40-75, 3 | 3420 | 8961 | 23 | 7 | 23 | 10 | 135 | 50.0 | 58.5 | 0.11 | 2.81 |
| RS model, 40-80, 1 | 5509 | 14572 | 20 | 6 | 26 | 12 | 152 | 56.4 | 68.5 | 0.18 | 4.31 |
| RS model, 40-80, 2 | 4439 | 11680 | 21 | 6 | 25 | 12 | 148 | 54.2 | 66.2 | 0.17 | 3.90 |
| RS model, 40-80, 3 | 3743 | 9804 | 22 | 6 | 24 | 11 | 140 | 51.8 | 63.2 | 0.13 | 2.86 |
| RS model, 45-70, 1 | 3756 | 9896 | 24 | 8 | 22 | 10 | 130 | 48.1 | 55.4 | 0.12 | 2.80 |
| RS model, 45-70, 2 | 3029 | 7928 | 25 | 8 | 21 | 9 | 122 | 45.4 | 52.0 | 0.10 | 2.42 |
| RS model, 45-70, 3 | 2563 | 6670 | 26 | 9 | 20 | 9 | 119 | 43.8 | 51.0 | 0.09 | 2.25 |
| RS model, 45-75, 1 | 4321 | 11390 | 22 | 7 | 24 | 11 | 138 | 52.6 | 62.5 | 0.14 | 3.17 |
| RS model, 45-75, 2 | 3488 | 9133 | 23 | 7 | 23 | 10 | 129 | 49.3 | 58.9 | 0.12 | 2.66 |
| RS model, 45-75, 3 | 2947 | 7675 | 24 | 8 | 22 | 10 | 126 | 48.2 | 57.1 | 0.10 | 2.50 |
| RS model, 45-80, 1 | 4795 | 12641 | 21 | 6 | 25 | 12 | 141 | 54.3 | 67.0 | 0.17 | 3.60 |
| RS model, 45-80, 2 | 3872 | 10140 | 22 | 6 | 24 | 11 | 134 | 52.3 | 64.0 | 0.13 | 2.77 |
| RS model, 45-80, 3 | 3269 | 8517 | 23 | 7 | 23 | 11 | 130 | 50.5 | 61.5 | 0.11 | 2.61 |
| RS model, 50-70, 1 | 3047 | 7982 | 26 | 8 | 21 | 9 | 114 | 44.8 | 52.4 | 0.10 | 2.31 |
| RS model, 50-70, 2 | 2459 | 6393 | 26 | 9 | 20 | 9 | 111 | 43.0 | 50.6 | 0.08 | 1.99 |
| RS model, 50-70, 3 | 2083 | 5386 | 28 | 9 | 19 | 8 | 104 | 40.5 | 47.8 | 0.09 | 1.98 |
| RS model, 50-75, 1 | 3612 | 9475 | 24 | 7 | 23 | 10 | 122 | 49.2 | 59.4 | 0.11 | 2.65 |
| RS model, 50-75, 2 | 2917 | 7601 | 24 | 7 | 22 | 10 | 118 | 47.5 | 57.2 | 0.09 | 1.96 |
| RS model, 50-75, 3 | 2469 | 6398 | 26 | 8 | 21 | 9 | 110 | 44.8 | 53.4 | 0.10 | 2.39 |
| RS model, 50-80, 1 | 4084 | 10723 | 23 | 6 | 24 | 11 | 126 | 51.4 | 63.2 | 0.13 | 2.69 |
| RS model, 50-80, 2 | 3302 | 8609 | 23 | 7 | 23 | 11 | 123 | 49.5 | 61.4 | 0.11 | 2.19 |
| RS model, 50-80, 3 | 2790 | 7238 | 24 | 7 | 22 | 10 | 115 | 47.2 | 58.7 | 0.12 | 2.41 |

**Abbreviations:** COL, colonoscopy; CRC, colorectal cancer; LYG, life-years gain; FIT, fecal immunochemical test; NCPCS, National Colorectal Polyp Care score; RS, Risk-stratification.
